# Microbial tapestry of the Shulgan-Tash cave (Southern Ural, Russia): influences of environmental factors on the taxonomic composition of the cave biofilms

**DOI:** 10.1186/s40793-023-00538-1

**Published:** 2023-11-21

**Authors:** Natalia Gogoleva, Olga Chervyatsova, Alexander Balkin, Lyudmila Kuzmina, Elena Shagimardanova, Daria Kiseleva, Yuri Gogolev

**Affiliations:** 1 Research Department for Limnology, Mondsee, Universität Innsbruck, Mondsee, 5310 Austria; 2 Institute of Fundamental Medicine and Biology, Kazan Federal University, Kazan, 420111 Russia; 3 State Nature Reserve “Shulgan-Tash”, Irgyzly, 453585 Russia; 4 Institute for Cellular and Intracellular Symbiosis, Ural Branch of the Russian Academy of Sciences, Orenburg, 460000 Russia; 5 Ufa Institute of Biology, Ufa Federal Research Center, Russian Academy of Sciences, Ufa, 450054 Russia; 6 Loginov Moscow Clinical Scientific Center, Moscow, 111123 Russia; 7 Institute of Geology and Geochemistry, Ural Branch of the Russian Academy of Sciences, Ekaterinburg, 620016 Russia; 8 Institute of Fundamental Education, Ural Federal University named after the first President of Russia B.N. Yeltsin, Ekaterinburg, 620002 Russia; 9 Kazan Institute of Biochemistry and Biophysics, Federal Research Center “Kazan Scientific Center of the Russian Academy of Sciences”, Kazan, 420111 Russia

**Keywords:** Karst cave, Biofilm, Shulgan-Tash, 16S rRNA gene, *Crossiella*, *Nitrosococcaceae* wb1-P19, Ga0077536, Paleolithic painting

## Abstract

**Background:**

Cave biotopes are characterized by stable low temperatures, high humidity, and scarcity of organic substrates. Despite the harsh oligotrophic conditions, they are often inhabited by rich microbial communities. Abundant fouling with a wide range of morphology and coloration of colonies covers the walls of the Shulgan-Tash cave in the Southern Urals. This cave is also famous for the unique Paleolithic painting discovered in the middle of the last century. We aimed to investigate the diversity, distribution, and potential impact of these biofilms on the cave’s Paleolithic paintings, while exploring how environmental factors influence the microbial communities within the cave.

**Results:**

The cave’s biofilm morphotypes were categorized into three types based on the ultrastructural similarities. Molecular taxonomic analysis identified two main clusters of microbial communities, with *Actinobacteria* dominating in most of them and a unique “CaveCurd” community with *Gammaproteobacteria* prevalent in the deepest cave sections. The species composition of these biofilms reflects changes in environmental conditions, such as substrate composition, temperature, humidity, ventilation, and CO_2_ content. Additionally, it was observed that cave biofilms contribute to biocorrosion on cave wall surfaces.

**Conclusions:**

The Shulgan-Tash cave presents an intriguing example of a stable extreme ecosystem with diverse microbiota. However, the intense dissolution and deposition of carbonates caused by *Actinobacteria* pose a potential threat to the preservation of the cave’s ancient rock paintings.

**Supplementary Information:**

The online version contains supplementary material available at 10.1186/s40793-023-00538-1.

## Background

Caves represent specific biotopes characterized by stable, typically low temperatures, high humidity, and scarcity of organic substrates. The absence of light imposes restrictions on primary productivity, which either absent [1] or associated with chemoautotrophs, such as ammonium, nitrite, hydrogen sulfide, methane, and manganese-iron oxidizers [2–5]. These conditions form special microbial communities consisting predominantly of psychrophilic oligotrophs [6, 7]. Although these communities are often very diverse, they are still poorly understood, making them a source of new species, genera and families of bacteria and archaea [7–10].

Throughout history, people have visited and used caves for various purposes. Currently, growing tourist interest is increasing the anthropogenic pressure on these fragile ecosystems, which makes it important to comprehensively monitor and study cave microflora. The Shulgan-Tash cave, located in the foothills of the Southern Urals, is formed within a carbonate karst massif consisting of limestones from the Upper Devonian and Lower Carboniferous periods [9]. The cave is known due to the discovery of a Paleolithic rock art monument, which today is the easternmost in Europe [10, 11]. Currently, more than 200 wall drawings dating back to the Upper Paleolithic, 20,600–16,500 years ago, have been described in the four halls of the cave [10, 12–14]. Most of the drawings were made with red ocher, a natural mineral pigment in which hematite predominates [14]. For many years the cave was subjected to uncontrolled anthropogenic pressure and suffered from vandalism. At the same time some biofilm formations also cover portions of the panels, nearing the ancient rock paintings. The potential danger of this fouling for paintings has not yet been assessed. Meanwhile, the possibility of this threat has been repeatedly shown previously [15, 16]. Previous studies have shown that microbial biofilms, such as the “Cave silver,“ can be found worldwide in underground cavities within rocks of various lithologies [17–31]. This wide occurrence may be attributed to the specific morphology of the cave, as well as its microclimatic and hydrological conditions [18–27]. The Shulgan-Tash cave has many features of microclimate and geology and is geographically remote from the studied communities. It seems interesting to find out the influence of these factors on the structure and functionality of similar biofilms. In addition, this rather extensive cave has a wide range of its own biotopes, differing both in conditions and morphology of fouling [32, 33], some of which have not previously been studied in detail.

In this work, we studied the morphological diversity of various biofilms on the walls of the Shulgan-Tash cave using cytological methods, and also carried out genetic barcoding to describe their taxonomic composition. The work presents data from long-term microclimatic observations and studies of the chemical composition of substrates. Correlation analyzes were conducted to understand how environmental factors, including microclimate parameters, distance from entrance, and substrate chemistry, influenced the taxonomic and metabolic characteristics of the biofilms studied. Particular attention is paid to identifying factors contributing to the abundant bacterial colonization of certain areas of the cave. In addition, we examined the role of bacteria in the dissolution and deposition of calcium carbonate (CaCO_3_) on rock surfaces in an effort to understand the potential biological threat they pose to the rock art.

## Materials and methods

### Cave site description

The Shulgan-Tash Cave (also known as Kapova Cave) is located on the territory of Shulgan-Tash Nature Reserve (53°02’ N, 57°03’ E), in the Tirmentau massif (height of the massif is 420 m.a.s.l., the entrance to the cave is 280 m.a.s.l.). The area is located in the western foothills of the Southern Urals, in the mountain-forest zone, at an altitude of 400–600 m.

**Fig. 1:**
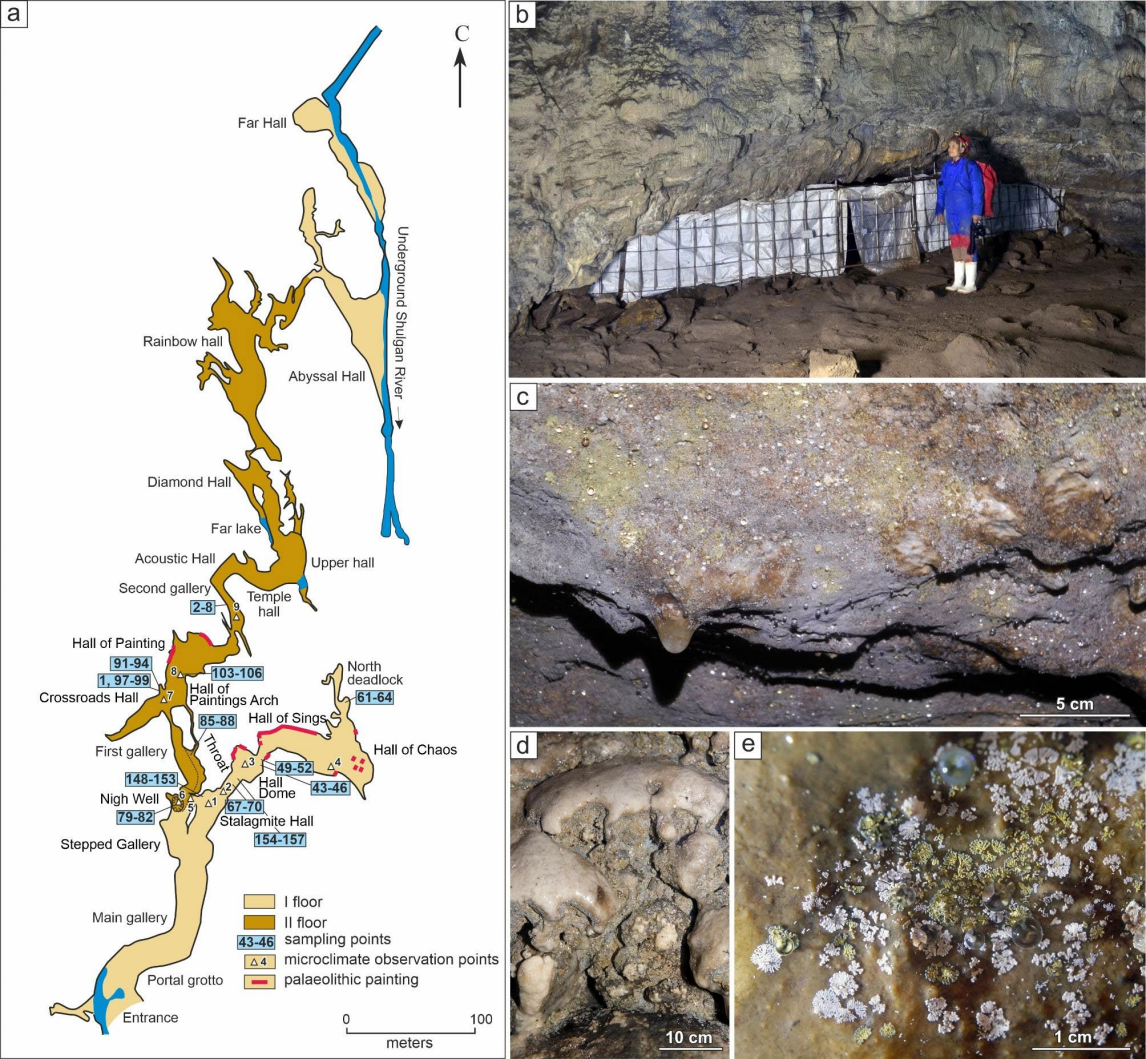
Cave map and sampling sites: (**a**) Map of the Shulgan-Tash Cave (Modifed from [37]). The sampling sites are indicated in the blue boxes; (**b**) the biofilms on the ceiling of the Throat passage; (**c**) continuous epilithic fouling coverings on the ceiling of the Arch in the Hall of Painting with abundant condensation; (**d**) continuous epilithic fouling on speleothems in the Stalagmite Hall; (**e**) macrophotography of individual biofilms

The Shulgan-Tash cave is situated in the temperate continental climate zone with excessive moisture (Dfb according to the Köppen-Geiger classification). The average long-term air temperature (MAT) in the region is + 0.9 °C. The maximal average monthly temperatures are observed in July (+ 16.8 °C), the minimal in January (–15.9 °С), the amount of precipitation varies from 365.5 mm per year to 901.2 mm per year. Geologically, the area of the Shulgan-Tash cave is located within the miogeosynclinal Paleozoic framing of the Bashkir meganticlinorium. The cave was laid out in gray pelitomorphic and organogenic limestones of Devonian and Lower Carboniferous age [9]. The cave is a two-level system of karst cavities with a length of 3323 m, an amplitude of 165 m and the presence of deep (up to − 80 m) siphon channels of the phreatic zone (Fig. 1a). The cavities of the first (lower level) floor are located at the heights of 280–300 m.a.s.l., and the cavities of the second (upper level) floor are located at the heights of 330–400 m.a.s.l. The cavities of the cave lie at a depth of 68 − 15 m from the surface. The cave opens by a large entrance (36 × 21 m), which creates an illuminated zone up to 60 m from the entrance, populated by phototrophic organisms - angiosperms, ferns, mosses, algae and cyanobacteria [32, 34, 35]. Nine species of bats hibernate in the cave, but the number of specimens does not exceed several hundred [36]. There is a 373 m long excursion route from the entrance to the Stalagmite Hall and the Stepped Gallery), which is visited by 65,000–70,000 people per year. Visiting the internal cavities of the cave (further than the Throat and the Near Well) is limited to 100–300 people per year.

Microbial biofilms develop on the walls and ceiling of the aphotic part of the cave, at a distance of 120 to 470 m from the entrance. The highest density of colony formation (up to n × 100 individual colonies per 10 cm^2^) is observed in the areas of the intersecting air flow patterns (Stalagmite Hall, Throat, etc.) causing active condensation processes (Fig. 1b, c). The biofilms can consist of separate white, greenish, gray colonies (Fig. 1e), or they are formed by continuous epilithic fouling (Fig. 1c, d).

### Microclimatic observations

Measurements in the cave were carried out according to the 8-term system every 3 h (0, 3, 6, 9, 12, 15, 18 and 21 h). The Testo 425 anemometer (Testo, Germany) was used to monitor the dynamics of air flows in the cave. Monitoring of temperature (T) and relative humidity (RH) in the cave was carried out in 2017–2019 using data loggers Onset HOBO U23 Pro v2 (Onset Computer Corporation, Bourne, MA, United States). The Dräger X-am 7000 GOST gas analyzer (Dräger, Lübeck, Germany) was used to observe the CO_2_ content. Air samples for the CO_2_ carbon isotopic analysis were taken in vials (Labco Exetainer, Lampeter, Wales, United Kingdom). The analysis was performed on the Delta V isotope ratio mass spectrometer (Thermo Scientific, Waltham, MA, United States) in the Institute of Geology, Innsbruck University, Austria. The results of ^13^ C analyzes were normalized to the V-PDB standard.

### Geochemical analysis of substrates and drip waters

Determination of the chemical composition of the rocks was carried out after preliminary grinding and pressing into pellets with polyvinyl alcohol by X-ray fluorescence analysis (XRF) on the multichannel X-ray spectrometer SRM-35 (Nauchpribor, Russia) and the energy-dispersive X-ray spectrometer EDX-8000 (Shimadzu, Japan) in the “Geoanalitik” shared research facilities of the Institute of Geology and Geochemistry of Ural Branch of RAS (Yekaterinburg, Russia). Loss on ignition (LOI) and thermal characteristics of rocks were determined on a synchronous thermal analyzer STA 449 F3 Jupiter (Netzsch, Germany) at the temperature range of 40–110 °C.

Determination of nitrogen and phosphorus compounds in dripping water was carried out by photocolorimetric method; for NO_2_ and NO_3_ with Griess reagent (for NO_3_ after reduction to NO_2_) with a technical error of about 10%. Monitoring was carried out from 2015 to 2020 at 10 drip points in the biofilm zone, and at 6 drip points outside the zone, in the far halls.

### Electron microscopy and local EDX analysis

Electron microscopic studies and semi-quantitative analysis of the elemental composition of the biofilm and substrate samples were carried out at the Institute for Metals Superplasticity Problems, RAS (Ufa, Russia). The measurements were performed using TESCAN Vega 3 and TESCAN Mira 3 instruments (TESCAN, Czech Republic) with an X-ACT EDX analyzer (Oxford Instruments, UK) in back-scattered electron (BSE) mode with Au-Pd sputtering according to the manufacturer’s instructions.

### Sampling, DNA extraction, PCR amplification and sequencing

The samples were collected in July of 2018 (Additional file 1: Fig. S1); the sampling sites are shown on Fig. 1a. The biofilm fragments were scraped off the walls with a sterile scalpel and placed in the RNA-later (Invitrogen, USA) solution. The samples were transported to the laboratory and stored at − 24 °C until DNA extraction.

DNA was extracted by the DNeasy PowerBiofilm Kit (Qiagen, Germany) with FastPrep-24 homogenizer (MP Biomedicals, USA) according to the protocol provided by the manufacturer. The amount of DNA was assessed using Qubit (Invitrogen, USA) and Qubit dsDNA High Sensitivity Kit (Invitrogen, USA). Bacterial V4 variable regions of the 16S rRNA genes were amplified with the universal primers 515F (5’-GTGCCAGCMGCCGCGGTAA-3’) and 806R (5’-GGACTACHVGGGTWTCTAAT-3’). Each 806R primer contained different barcodes [38]. Archaeal V3 to V4 variable regions of 16S rRNA genes were amplified with the universal primers Arch349F (5’-GYGCASCAGKCGMGAAW-3’) and Arch806R (5’-GGACTACVSGGGTATCTAAT-3’) [39].

A negative sterile water control was always performed to confirm the absence of DNA contamination. The libraries containing bacterial 16S rRNA genes were sequenced using MiSeq v2 Reagent Kit 500 cycles (Illumina, USA), the libraries containing archaeal 16S rRNA genes were sequenced using MiSeq v3 Reagent Kit 600 cycles (Illumina, USA) on the MiSeq platform (Illumina, USA). All DNA manipulations and sequencing were performed at Joint KFU-Riken Laboratory, Kazan Federal University (Kazan, Russia).

### Data analysis

Potential primers and adapter sequences were screened and removed using a bbduk program (https://sourceforge.net/projects/bbmap/). Trimmed sequences were processed using the DADA2 pipeline [40], including filtering by quality and length, denoising, chimera removing, and Amplicon Sequence Variants (ASVs) recovering. Taxonomy assignment was done using the RDP classifier [41] with 50% confidence threshold [42] against the Silva prokaryotic SSU taxonomic database (version 138.1).

Count table and taxonomy annotation of amplicon sequence variants (ASVs) were treated with phyloseq [43], vegan [44], ggplot2 [45], reshape [46] and dependent packages for graphical representation and statistical analysis, including calculation Chao1 and Simpson indices to display alpha richness and alpha diversity of samples, respectively. Centered log ratio (CLR) transformation was applied for data normalization. Non-metric Multi-dimensional Scaling (NMDS) with Bray-Curtis dissimilarities [47] was used for beta diversity assessment.

Differentially abundant bacterial taxa between microbial communities were identified using linear discriminant analysis effect size (LefSe) on the cumulative sum scaling normalized data [48]. KW tests along with linear discriminant analysis (LDA > 2) were used to determine differentially abundant features using the lefser package [49].

Core microbiome was analyzed at 50% sample prevalence and 0.01% relative abundance cut-off values with microbiome R package [50]. Permutational multivariate analysis of variance was used to test correlations between community composition and sample characteristics. Statistical processing of microclimatic and hydrochemical data, as well as CCA analysis, were performed using the XLSTAT 2020 and PAST 4.0 programs [51].

For the 16S rRNA gene phylogenetic analysis, the closest relatives were recovered by BLAST searches against 16S rRNA gene sequence database, additional sequences corresponding to the best BLAST hits (non-redundant), and sequences of the type strains were added, and the sequence of *Egibacter rhizosphaerae* (NR_147752.1) was used as the outgroup. Sequences were aligned using the ClustalW algorithm and trimmed to the amplicon sequence length (254 bp) [52].

The phylogenetic tree was calculated by rapid bootstrap analysis of RAxML v8.2.11 [53] (100 pseudoreplicates) under the model GTR + I + 0 and the maximum-likelihood algorithm. The resulting V4 16S rRNA gene trees were edited in Interactive Tree Of Life (iTOL) tool [54].

### Picrust functional prediction

Functional prediction of the Shulgan-Tash cave microbial community was provided by PICRUSt2 program based on the results of the 16S rRNA gene amplicon sequencing [55]. Representative set of the dominant ASVs in the community (with a minimum representation of > 0.01% of all reads in at least a single sample) was taken into analysis. MetaCyc and KEGG databases were used for functional prediction. Visualization of the predicted metabolic pathways and comparison across biofilms were provided in the STAMP program [56]. Multiple pairwise comparison of the samples provided with Tukey-Krammer test [57]. Significant differences in metabolic pathways across samples were determined by ANOVA followed by Tukey-Krammer test (*p* < 0.05).

### Data availability

The 16S rRNA gene sequencing data have been submitted to the NCBI’s Sequence Read Archive (SRA) under the accession number BioProject PRJNA1018771.

## Results

### Morphology and localization of biofilms

**Fig. 2:**
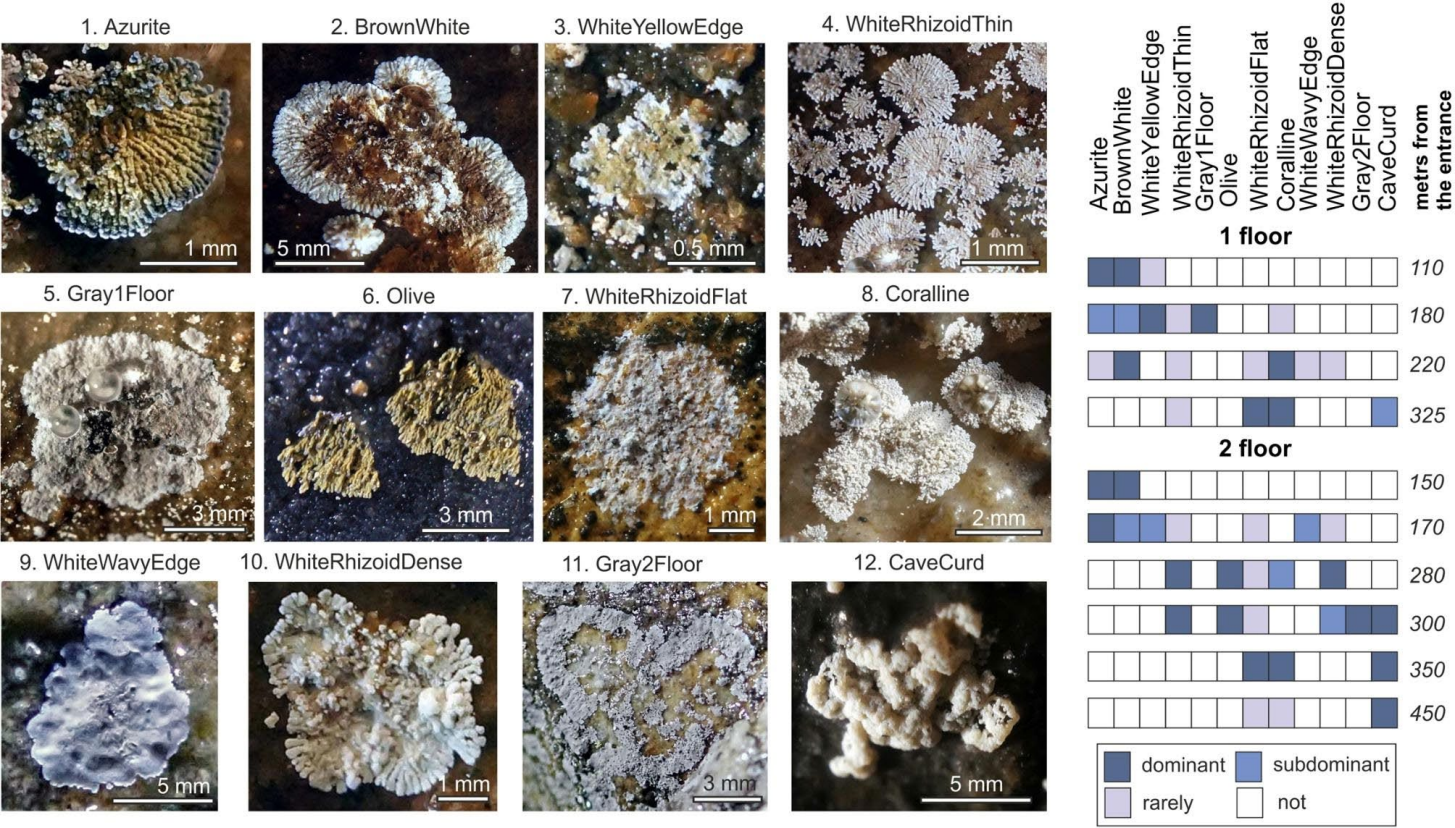
Morphology of individual colonies and localization of the biofilm morphotypes along the Shulgan-Tash cave

Abundant continuous microbial fouling was observed in the near aphotic part of the cave in the zone from 120 to 300 m from the entrance (Figs. 1 and 2; Additional file 2) with active air drift from the surface (Additional file 1: Fig. S2). Some fluctuations in air temperature and humidity were noted in this zone (Additional file 1: Fig. S3). In the distant zones (325 m on the 1st floor, 300–450 m on the 2nd floor), characterized by slight temperature fluctuations, the density of biofilm development decreased and corresponded to individual colonies by several dozens of cm^2^. Further 470 m from the entrance, where the temperature and humidity remained constant, there was no detected fouling. The diameter of individual colonies, biofilm structure and color were used to classify the wall microbial fouling. We identified 12 biofilm morphotypes named according to their appearance (Fig. 2; Additional file 2). The diameter of the colonies ranged from 1 to 2 mm (“WhiteYellowEdge”) to 10–30 mm (“CaveCurd”). According to the structure, individual colonies were subdivided into three-dimensional and flattened (hereinafter: 3d and 2d-types). The 3d-type colonies were represented by ramose, cheesy-microgranular and coral-like forms. The 2d-type colonies were represented by leaf-shape (with zigzag wavy edges) and fold-leaf-like forms (Fig. 2). The biofilms were formed by the colonies showing a variety of colors: white or light cream (5 types), two-tone white-brown, white-yellow and blue-green (3 types); colonies with homogeneous or gradient olive and gray colors (4 types)

**Fig. 3:**
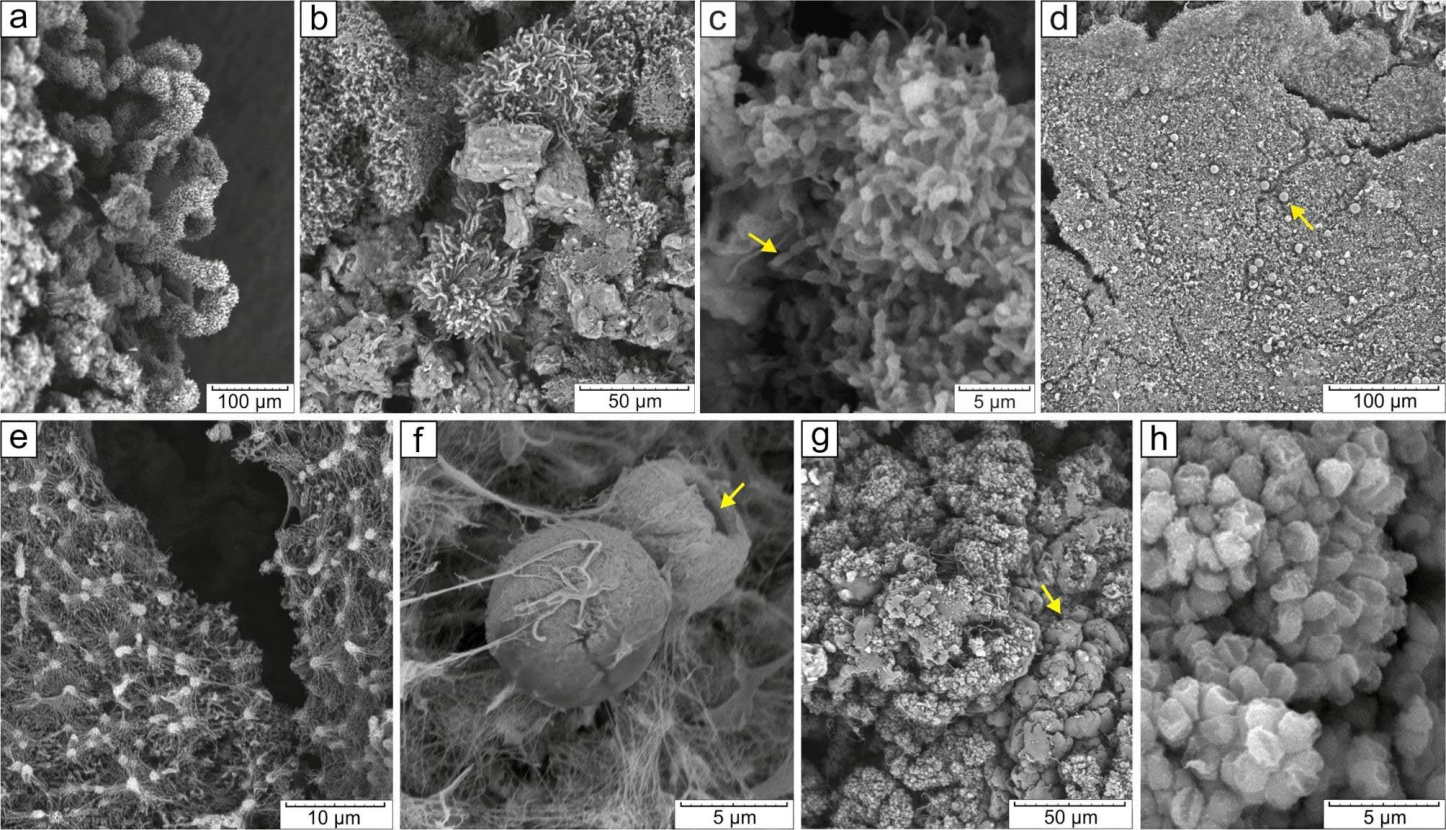
Scanning electron microscope images of the predominant microstructures of the colonies: ***Structure №1***: **a, b**) sporangial-like tufts in a colony of an “Olive” morphotype; **c**) accumulation of spore chains in a “WhiteWavyEdge” colony, thickenings at the ends of branches shown by the arrow. ***Structure №2***: **d**) general view of a “BrownWhite” morphotype colony (spherical elements shown by the arrow); **e**) reticular structures with nodules in a “WhiteRhizoidFlat” morphotype colony; **f**) spherical elements in a “BrownWhite” morphotype colony (the arrow indicates a cavity). ***Structure №3***: **g**) mass of coccoid cells forming the biofilm “CaveCurd”; **h**) close-up of coccoid cells

“Azurite”, “WhiteYellowEdge”, “WhiteRhizoidThin”, “Coralline”, “Olive”, “WhiteRhizoidDense” biofilm morphotypes were formed by single colonies and continuous epilithic microbial fouling, while “BrownWhite”, “WhiteWavyEdge”, “CaveCurd”, “Gray1Floor”, and “Gray2Floor” morphotypes were formed only by single colonies. Some morphotypes were strictly localized in certain cave zones (Fig. 2; Additional file 1: Fig. S4; Additional file 2). “Azurite” and “BrownWhite” were found at a distance of 110 to 230 m from the cave entrance. “Gray1Floor” and “Gray2Floor” were localized on the ceilings in areas with abundant water condensation, such as the Throat passage areas with abundant condensation on the ceilings (185–195 m from the entrance) and in the Arch of the Hall of Painting (300–320 m from the entrance). “CaveCurd” was found only in the remote parts of the Cave, (290–325 m from the entrance on the 1st floor, and 300–450 m from the entrance on the 2nd floor). The colored morphotypes were more prevalent in the near proximal part of the cave, while the colorless ones gradually replaced them with distance from the entrance (Fig. 2). We identified three main types of structural organization of the colonies (Fig. 3). Biofilms of various morphotypes could include one structural type or combine several of them.

***Structure №1*** was typical for the biofilms with ramose and coral-like 3d-morphology (“Azurite”, “WhiteWavyEdge”, “WhiteRhizoidThin”, “Coralline”, “Olive”, “WhiteRhizoidDense”). The colonies formed branching filamentous structures (Fig. 3a, b) with spherical thickenings 0.7-1 μm in size on the filaments (Fig. 3c, indicated by the arrow).

***Structure №2*** Structure 2 was a network of filaments several nanometers thick with nodules and loops (Fig. 3e), in which spherical elements 1–9 μm in diameter were embedded (Fig. 3d, indicated by the arrow, f). Some spherical elements had internal cavities (Fig. 3f, indicated by an arrow). This structure was typical for the 2d-type biofilms (“BrownWhite”, “WhiteRhizoidFlat”, “Gray1Floor”, “Gray2Floor”).

***Structure №3*** was dominant for the “CaveCurd” morphotype. It was a shapeless mass of coccoid cells about 1.2 μm in size (Fig. 3h) covered with remnants of a filamentous matrix (Fig. 3g) (probably exopolysaccharide matrix (EPS)).

The 3d-morphotypes developed mainly on slightly moistened substrates, where water was present only as film or capillary solutions. As a rule, the biofilms of these types had weak attachment to the substrate, with the exception of the “BrownWhite”. The 2d-morphotypes (with the exception of the “WhiteRhizoidFlat”, which preferred relatively dry conditions) advanced in humid conditions, often in places where karst waters were running off. These biofilms were well attached to the substrate. For the “WhiteYellowEdge” morphotype SEM studies have not been carried out.

### Microclimatic conditions in the Shulgan-Tash cave

The results of three years of observations indicate that the air exchange within the Shulgan-Tash cave is different in summer and winter. Between May and September, the density of the warm outside air is lower than the average air density within the cave. In November, there is a transition to the winter regime where the ratio of air densities becomes reversed, and this continues until the end of March. As a result, bidirectional convective circulation always occurs within the cavities of the 1st floor and this reverses with the season (Additional file 1: Fig. S2). In the cavities of the 2nd floor, there are unidirectional air currents that move inside the cave during winter and outside in summer. This indicates that the channels that communicate with the soil and the epikarst are involved in the air exchange process [58–60]. Fig. S3 (Additional file 1) presents the results of temperature (T), relative air humidity (RH), and CO_2_ content at some key points in the cave.

The area with the highest amplitude of seasonal temperature fluctuations is located within 120 m from the entrance, which includes the Stalagmite Hall and the Throat Passage. In this part of the cave there is good air exchange with the surface, the most active in summer (Additional file 1: Fig. S2). Temperature fluctuations on the surface can be felt at a distance of up to 250 m from the entrance on the 1st floor and up to 300 m on the 2nd floor. In the more distant part of the cave, air temperatures range from 7.8 to 6.1 °C. The areas where biofilm develops coincide with the regions experiencing positive temperatures. Relative humidity (RH) fluctuations within measurement accuracy (RH < 98%) were observed only at the inlet (Additional file 1: Fig. S3). The absolute humidity of the air ranged from 7 to 8 g/m^3^. During the summer, when warm and humid air enters the cave, significant condensation of water vapor occurs on the surface of the vaults and walls (Additional file 1: Fig. S5).

During the winter circulation mode, the CO_2_ content on both floors of the cave was similar to atmospheric levels (0.03–0.04% vol.). However, during the summer circulation regime, the CO_2_ content in the cavities of the 1st floor slightly increased to 0.04–0.05% vol., while in the cavities of the 2nd floor, particularly in the Hall of Figures, the CO_2_ concentration significantly rose to 0.35–0.45% vol. The carbon isotope composition of CO_2_ in the air of the remote part of the 2nd floor ranged from *δ*13C = -19.0 to -21.5‰ VPDB, with *n* = 4 and a standard deviation of 1.2‰. These values are typical for air in caves where CO_2_ is generated from soil and vadose zone air [61–65].

### Chemical composition of substrates and infiltration water

Most of the biofilms described above were able to colonize all available substrates in the cave, but they were mainly found on limestone that had been altered due to condensation corrosion. In this case, under the action of condensation water, intergranular dissolution of limestone occurs (micritization), and the surface layer of the wall turns into a microgranular gel suspension several millimeters thick. Speleothems (flowstone, coralloids, more rarely moonmilk) and non-altered limestone were also utilized by biofilms. Some types of the biofilms were found only on certain types of substrates. “BrownWhite” was localized on hard sintered flowstone, “Gray1Floor” and “Gray2Floor” colonized micritized limestone. “Coralline” tended to substrates rich with clay minerals. The results of the chemical analysis of different substrates are provided in Additional file 1: Table S1.

Since the main mineral component of limestone and speleothems is calcite, CaO was the major registered compound due to the thermal decomposition of CaCO_3_ during ignition, while CO_2_ was removed. Correlation analysis (Spearman correlation, *p* < 0.05) and detrended correspondence analysis (DCA) revealed the following compound associations (Additional file 1: Fig. S6): (1) calcite – CaO and CO_2_ ignition loss; (2) phyllosilicates – Al_2_O_3_ and SiO_2_ as major components, TiO_2_, MgO, Fe_2_O_3_, K_2_O, and Na_2_O [66]; (3) biogenic association including P_2_O_5_ and S. Phosphorus and sulfur are presumably associated with organic matter mineralization and biological erosion of substrates [67]. The content of MnO, one of the biologically important compounds, was below the analytical error in most substrates. The substrate most enriched with phyllosilicates was found under the “Coralline” biofilm. The substrates under the “WhiteWavyEdge”, “WhiteYellowEdge”, and “Gray1Floor” biofilms were most enriched with elements of the biogenic association. The substrate with the fewest impurities was characteristic of the “CaveCurd” biofilm background.

We monitored the concentration of NO_3_ and NO_2_ in drip waters in different seasons (Additional file 1: Table S2, 3). The average content of NO_3_ in the waters of the halls with the biofilms was 4.1 mg/L (*n* = 201), while in the halls located at a distance of more than 450 m from the entrance, where no visible microbial growth was observed, it was only 0.7 mg/L (*n* = 34). The average NO_2_ concentration was 0.05 mg/L (*n* = 196) and 0.01 mg/L (*n* = 32), respectively. The found differences in the content of NO_3_ and NO_2_ are statistically significant (Kruskal-Wallis test, *n* < 0.01).

### Analysis of taxonomic diversity of the biofilm-forming communities

#### Taxonomic composition of bacteria and archaea

**Fig. 4:**
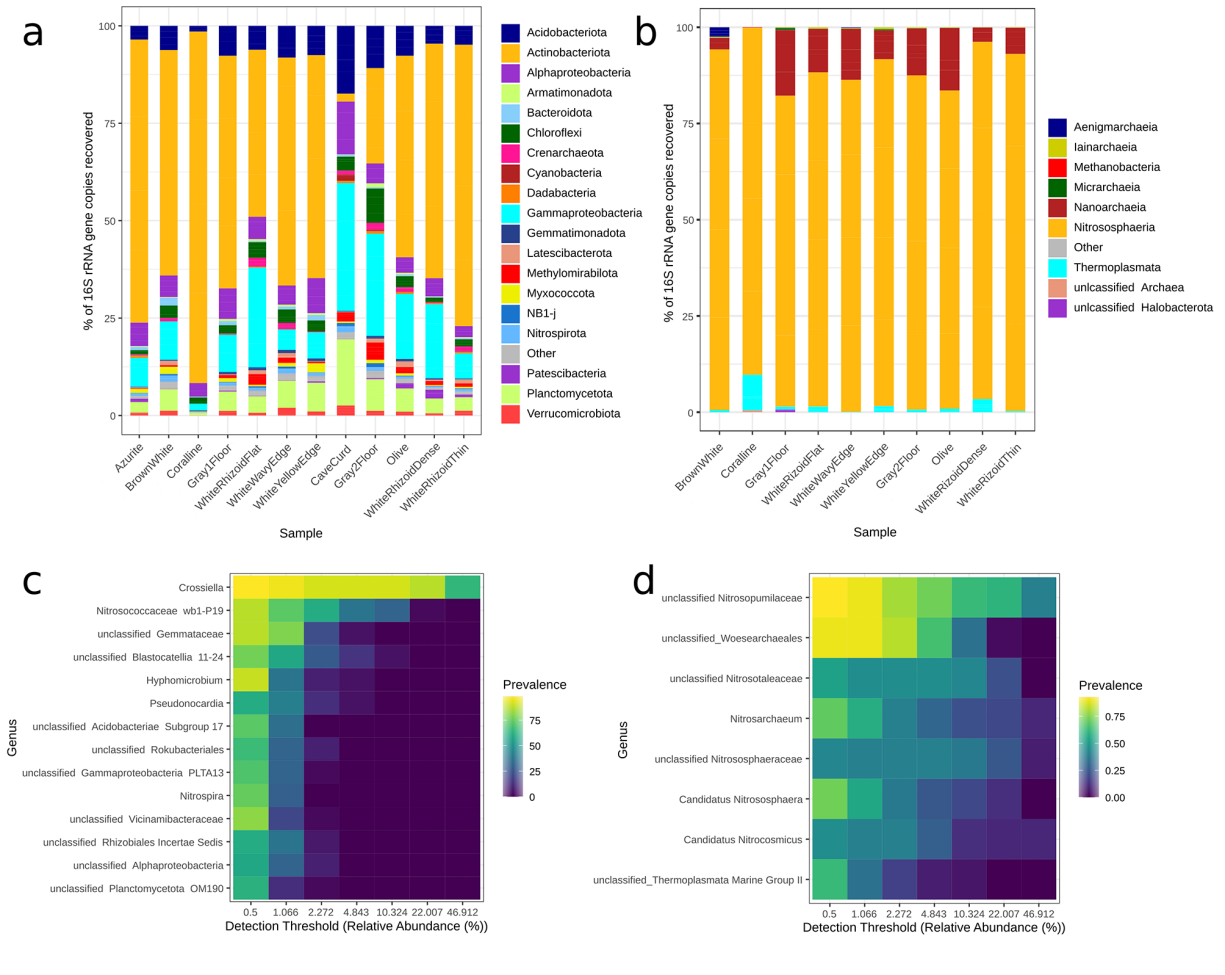
**a**, **b** – Relative abundances of the bacterial and archaeal phyla in the communities of the cave biofilms (phylum *Proteobacteria* is subdivided into classes). Only the phyla with the abundance of ≥ 1% in the analyzed samples are present; the other phyla are merged into the “Other” category. Classification of the ASVs was conducted against the Silva database (138.1 release) with 50% threshold. c, d – Core microbiome analysis based on relative abundance and sample prevalence of the bacterial and archaeal genera taxa in the biofilms of the Shulgan-Tash cave. Prevalence was assessed as ASV detected in the range from 50 to 100% samples

The taxonomic composition of all described microbial biofilms was determined by the 16S rRNA gene metabarcoding. The dominant bacterial phyla in the biofilms included *Actinobacteria*, *Proteobacteria*, *Acidobacteria*, *Planctomycetes*, and *Chloroflexi* (Fig. 4a). Representatives of the phylum *Actinobacteria* were the predominant organisms in the majority of the studied biofilms. However, in the “CaveCurd’’ and Gray2Floor samples *Proteobacteria* were the most abundant. *Acidobacteria* were also present in all studied biofilms.

A significant part (~ 74% of total ASVs) of cave microbiome consisted of the organisms that could not be classified to the genus level (Additional file 1: Fig. S7). “CaveCurd” and “Gray2floor” biofilms contained especially many such bacteria. The members of the *Pseudonocardiaceae* (*Actinobacteria*) family were found in all biofilms; there were up to 70% ASVs belonging to them in “Asurit”, “Coralline” and “Olive” biofilms.

For a more detailed study of the archaeal component, we prepared the libraries after amplification of the 16S rRNA gene region using archaea-specific primers (amplification of the samples of “Azurite” and “CaveCurd’’ did not give a result). The representatives of the phyla *Thaumarchaeota* (formerly *Crenarchaeota*) [68] and *Nanoarchaeota*, were the most abundant in the archaeal part of the microbial communities (Fig. 4b). Representatives of the phyla *Aenigmarchaeota*, *Euryarchaeota*, *Micrarchaeota*, and *Thermoplasmatota*, were also detected. Representatives of the phylum *Thaumarchaeota* dominated among archaea in all samples. The fraction of *Thaumarchaeota* usually made up more than 60% of the archaeal community, with the exception of “Gray1Floor” and “WhiteRizoidDense” biofilms where their share was less than 50%. No representatives of the phylum *Nanoarchaeota* were found in the “Coralline” biofilm, while in other samples this phylum composed from 1.38 to 12.26% of the archaeal community. Several dominant members of the phylum *Thaumarchaeota* have been reliably identified at the genus level, such as *Ca.* Nitrosoarchaeum, *Ca.* Nitrososphaera, and *Ca.* Nitrocosmicus (Additional file 1: Fig. S8). However, the majority of archaeal ASVs could be reliably assigned only to the taxa of the family (Additional file 1: Fig. S8, 9).

### Characterization of the core cave microbiome and dominant taxa of the cave biofilms

Core microbiome analysis revealed that 14 genera were shared among all 12 biofilm morphotypes, with a minimum detection threshold of 0.5% (Fig. 4c, d). Prevalence was determined based on the presence of the ASVs detected in 50– 99.9% of the samples. The core Shulgan-Tash cave microbiome was composed of the following taxa: *Actinobacteria* (*Crossiella, Pseudonocardia*), *Planctomycetota* (unclassified *Gemmataceae*, unclassified *Planctomycetota* OM190), *Alphaproteobacteria* (*Hyphomicrobium*, unclassified *Alphaproteobacteria*, and *Rhizobiales*), *Gammaproteobacteria* (*Nitrosococcaceae* wb1-P19, unclassified *Gammaproteobacteria*), *Nitrospira*, *Acidobacteria* (*Blastocatellia* 11–24, unclassified *Vicinamibacteria* and *Rokubacteriales*, *Acidobacteria* Subgroup 17).

*Actinobacteria* were predominant in all studied biofilms, except the “CaveCurd” biofilm where *Gammaproteobacteria* dominanted (Fig. 4a, c; Additional file 1: Fig. S10). The phylum *Actinobacteria* was represented by *Crossiella* (ASV1, ASV2, ASV3, and ASV8 assigned to the *Crossiella* genus with a high confidence level > 98%), *Pseudonocardia* (ASV49), *Kribbella* (ASV36), and unclassified *Euzebyaceae* (ASV24).

*Nitrosococcaceae* wb1-P19 was widely represented in the core cave microbiome (Fig. 4c). *Nitrosococcaceae* wb1-P19 and *Gammaproteobacteria* Ga0077536, both representatives of the *Gammaproteobacteria* class, were dominant taxa in the “CaveСurd”, “Gray2Floor”, “WhiteRhizoidFlat”, “Olive”, “WhiteRhizoidThin”, and “WhiteRhizoidDense” biofilms (Additional file 1: Fig. S10). Among the three most abundant sequences associated with this class, ASV4 and ASV5 were identified as *Nitrosococcaceae* wb1-P19.

Five ASVs were assigned to the phylum *Acidobacteria*. ASV7, ASV10, and ASV91 were identified at the genus level as *Blastocatellia* 11–24 with a confidence level of 100%. ASV25 was assigned to the order *Vicinamibacteria* Subgroup 17. The ASV42 was assigned to the as-yet-uncultivated clade RB41 of *Pyrinomonadaceae*. ASV13 was identified as an uncultured group *within* the *Rokubacteriales* order.

The *Gammaproteobacteria* Ga0077536 was represented by ASV6. ASV6 was predominant in the “CaveCurd” biofilm (Additional file 1: Fig. S10). Additionally, an unclassified member of the phylum *Chloroflexi*, specifically unclassified_*Anaerolineaceae* (ASV9), was present in the “Gray2Floor” and “WhiteRhizoidFlat” biofilms.

Phylogenetic analysis of the discovered actinobacterial ASVs revealed that they exhibit maximum sequence identity with the actinobacterial 16S rRNA genes found in other karst caves and lava tubes that harbor similar biofilms. It should be noted that all these *Actinobacteria* are non-cultivable (Additional file 1: Fig. S11). Similarly, representatives of the order Ga0077536 and the phylum *Acidobacteria* also demonstrated the highest sequence identity in their 16S rRNA genes with those from the biofilm samples from other caves (Additional file 1: Fig. S12, 13).

### Taxonomic structure and environmental impact

The alpha diversity of the cave biofilms was evaluated using the Chao-1 and Simpson indices (Additional file 1: Fig. S14). The “Gray1Floor”, “WhiteWavyEdge”, and “WhiteYellowEdge” biofilm samples showed the highest number of bacterial ASVs. The “Coralline” sample had the smallest number of bacterial ASVs, which were half the average for the studied samples. The Simpson index (Additional file 1: Fig. S14) indicated that the greatest diversity was observed in the “Gray2Floor” and “CaveCurd” biofilms, while the “Azurite”, “Coralline”, and “WhiteRhizoidThin” biofilms appeared to be less diverse. In terms of archaea, they were most abundant in the “Gray1Floor”, “WhiteYellowEdge”, and “Olive” biofilms. The “Coralline” biofilm sample had the fewest archaea ASVs. The highest archaeal diversity was found in the “Gray1Floor”, “WhiteYellowEdge”, and “WhiteRhizoidDense” biofilm samples. Conversely, the “WhiteRhizoidThin” biofilm sample exhibited the least diversity.

**Fig. 5:**
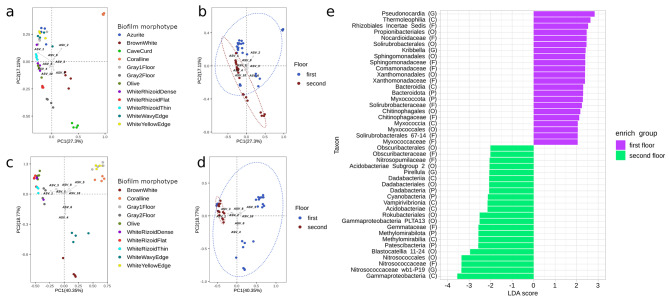
The PCA biplot of the bacterial (**a, b**) and archaeal (**c, d**) communities of the Shulgan-Tash cave was generated based on Bray-Curtis distance method. The points on the plot correspond to the individual samples. The plots b and c also included ellipses outlining the samples associated with a specific floor of the cave. **(e)** –The LEfSe analysis of the communities on the 1st and 2nd floors identified taxa with a linear discriminant analysis (LDA) score of 2.0 as the cut-off value. Abundant taxa on the 2nd floor were indicated with a negative LDA score (green), while abundant taxa on the 1st floor were indicated with a positive score (purple). The closest taxonomic-level identification of each taxon is indicated in parentheses, specifying whether it belongs to the phylum (P), class (C), order (O), family (F), or genus (G)

Principal Components Analysis (PCA) revealed distinct clustering of bacterial and archaeal communities based on sample types, with all replicates closely grouped together (Fig. 5a, b). The bacterial community of “CaveCurd” displayed the most pronounced uniqueness in terms of the composition of dominant taxa. The bacterial communities of “Coralline’’ and “BrownWhite” biofilms exhibited clear separation from other biofilms and from each other. *Actinobacteria*, specifically ASV2 and ASV3, dominated the “Coralline” and “BrownWhite” communities, respectively. In terms of archaeal communities, the samples from “Gray2Floor”, “Coralline”, and “WhiteYellowEdge” formed a separate cluster. The archaeal ASV5, corresponding to *Nitrososphaeraceae*, was more abundant in this cluster. ASV4 and ASV6 (*Nitrosarchaeum*) were associated with “BrownWhite” and “WhiteWavyEdge’’, respectively. The remaining communities showed a higher level of similarity and could not be clearly distinguished on the plot.

Based on the results of the PCA analysis, it was observed that the bacterial and archaeal communities clustered into two groups, corresponding to the 1st and 2nd cave floors (Fig. 5a, b). Notably, the “WhiteRhizoidFlat” samples taken from the 1st floor were grouped together with the clustered communities from the 2nd floor.

LEfSe analysis revealed 58 bacteria that were differentially abundant in the communities of the 1st and 2nd floors (Fig. 5e). The phyla that showed higher representation on the 1st floor were *Actinobacteriota*, *Myxococcola*, and *Bacteroidota*. On the 2nd floor, *Cyanobacteria*, *Acidobacteriota*, *Patescibacteria*, *Methylomirabilota*, *Planctomycetota*, and *Dadabacteria* exhibited higher representation. Specific representatives of *Proteobacteria* were differentially abundant on each floor. On the 1st floor, *Proteobacteria* were dominated by *Rhizobiales* Incertae Sedis, *Sphingomonadaceae*, *Comamonadaceae*, and *Xanthomonadaceae* families. On the 2nd floor, *Nitrosococcaceae* wb1-P19 and unclassified *Gammaproteobacteria *PLTA13 were prominent.

*Actinobacteriota* on the 1st floor were represented by *Kribella*, *Pseudonocardia*, unclassified *Solirubrobacterales 67 − 14*, and unclassified *Propionibacteriales*. The families *Chitinophagaceae* (*Bacteroidota*) and *Myxococcaceae* (*Myxococcota*) were also significantly more abundant in the biofilms of the 1st floor. Similarly, *Chitinophagaceae* (*Bacteroidota*) and *Myxococcaceae* (*Myxococcota*) were significantly more abundant in the 1st-floor biofilms. *Acidobacteriota*, which were significantly more abundant on the 2nd floor, were represented by *Blastocatellia* 11–24 and *Acidobacteriae* Subgroup 2 orders. *Dadabacteriales* (*Dadabacteria*) and *Rokubacteriales* (*Methylomirabilota*) were also more abundant in the biofilms of the 2nd floor. The representatives *Pirellula* and unclassified *Gemmataceae*, which were more abundant on the 2nd floor, showed a similar trend. The family *Obscuribacteraceae* represented the cyanobacteria. There was no correlation between biofilm color, structure, location, substrate under growing colonies, and sample clustering (as assessed by PERMANOVA, *p* = 0.05) (Additional file 1: Fig. S15).

**Fig. 6:**
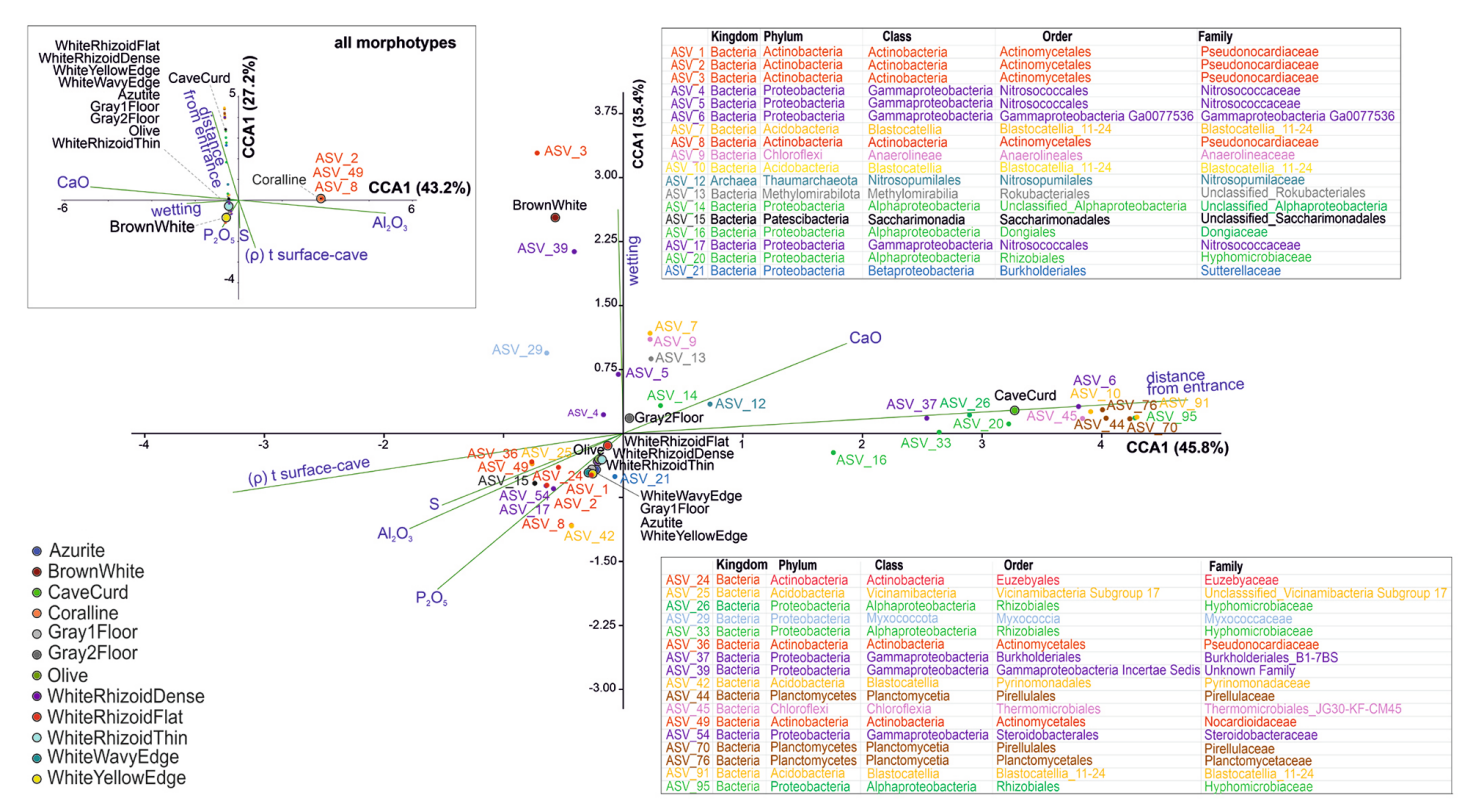
CCA ordination biplots showing dependence of the certain biofilms on the environmental factors. Top inset shows the initial variant including all morphotypes of biofilms; the main biplot was built without “Coralline” biofilm

Canonical correspondence analysis (CCA) [69] was conducted to assess the impact of topological, geochemical, and microclimatic factors on the taxonomic diversity of the investigated biofilms. Factors such as distance from the cave entrance, Spearman correlation coefficients between cave temperatures and surface temperatures (indicating the effectiveness of external atmosphere connection), and substrate moisture content (as shown in Additional file 2) were evaluated as topological and climatic factors. Geochemical factors included quantities of Al_2_O_3_, P_2_O_5_, S, and CaO in the substrates (as presented in Additional file 1: Table S1. Other compounds like Na_2_O, MgO, SiO_2_, K_2_O, TiO_2_, and Fe_2_O_3_ were not considered as they were closely correlated with Al_2_O_3_, being common components and additives of phyllosilicates. ASVs with a fraction of at least 1% in any of the biofilms were used as variables. Initially, the analysis was performed for all biofilms; however, it was observed that the ASVs corresponding to unknown members of *Pseudonocardiaceae*, which dominated in the “Coralline” biofilm, showed significant distance along the CCA1 axis. This discrepancy was likely due to the strong influence of the Al_2_O_3_ factor, as this biofilm exclusively develops on substrates rich in phyllosilicates. Consequently, a CCA plot without the “Coralline” biofilms was constructed and analyzed for more accurate scaling (Fig. 6). In both variants of the analysis, the variables demonstrated significant associations with environmental factors (permutation test, *p* = 0.02 for the first variant and *p* = 0.05 for the second variant).

Bacteria belonging to the class *Actinobacteria* tend to be more abundant in the near parts of the cave, which are influenced by the external climate. The distribution of *Actinobacteria* is correlated with the content of Al_2_O_3_, P_2_O_5_, and S, indicating their preference for substrates rich in phyllosilicates and biogenic elements.

Most *Gammaproteobacteria* in the biofilms are also found in the proximal part of the cave. Their distribution is associated with the “Wetting” factor, as they are primarily found in substrates that are abundantly moistened. Only one taxon of *Gammaproteobacteria*, specifically classified as the order *Nitrosococcales*, is characteristic of the far part of the cave, where it dominates in the “CaveCurd’’ biofilm associated with CaCO_3_-rich substrates.

Bacteria of the class *Alphaproteobacteria* are predominantly found in the distant parts of the cave, where the influence of the external climate is less significant. Bacteria of the class *Planctomycetes* are also restricted to the remote areas of the cave, as the biofilms enriched with these taxa tend to develop on chemically pure carbonate substrates, as indicated by their proximity to the CaO vector.

Archaea belonging to the family *Nitrosopumilaceae* are associated with well-moistened substrates composed of pure carbonate located in the remote part of the cave entrance.

Bacteria of the phylum *Methylomirabilota* are attracted to moist and pure carbonate substrates but within an environment with low concentrations of CO_2_. Bacteria of the phyla *Acidobacteria* and *Chloroflexi* do not demonstrate specific environmental preferences and can be found in diverse ecological niches. However, they are usually found in consortia with other microorganisms, likely being dependent on their metabolites [70, 71].

### Predicted functional composition of the Shulgan-Tash cave microbial community

The functionality of the microbial communities in the studied samples was predicted using PICRUSt2 analysis, KEGG, and MetaCyc databases. The abundance of pathways in each sample was determined based on these predictions.

**Fig. 7:**
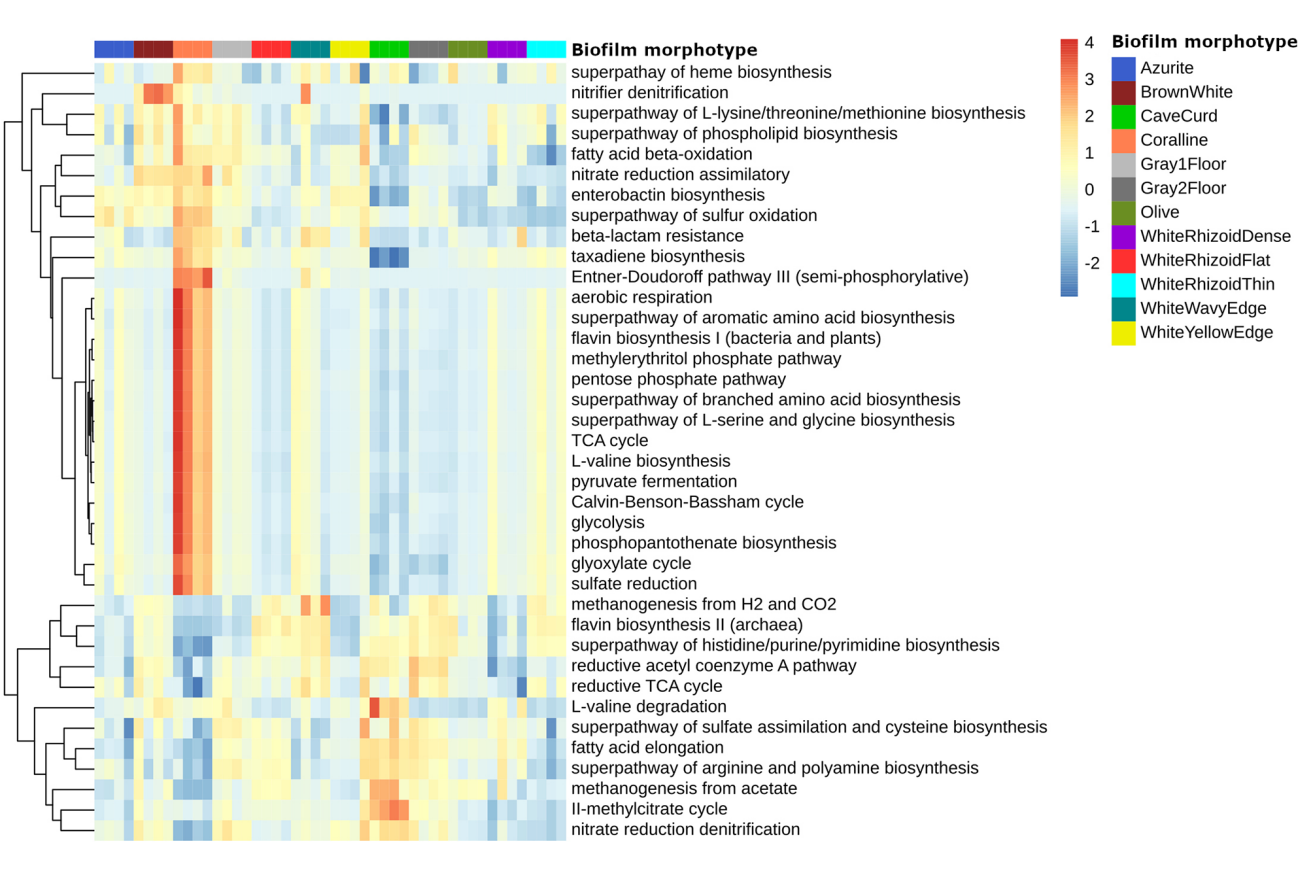
The heatmap of the selected MetaCyc metabolic pathways, predicted with PICRUSt2. Pathways clustered using Euclidian distance. The normalized relative abundance of each pathway is indicated by a gradient of color from blue (low abundance) to red (high abundance)

A total of 172 KEGG pathways and 382 MetaCyc pathways were predicted across all samples. Among them, 148 KEGG-predicted pathways and 361 MetaCyc-predicted pathways were detected in all analyzed samples (Additional file 3). Based on the KEGG predictions, the genes involved in amino acid metabolism (26.77%), carbohydrate metabolism (10.88%), nucleotide metabolism (10.21%), and energy metabolism (9.37%) were found to be prevalent in all samples. These pathways play crucial roles in the overall metabolic activities of the microbial communities. In terms of MetaCyc pathways, the most abundant metabolic pathways across all samples included aerobic respiration I, amino acid biosynthesis, pyruvate fermentation to isobutanol, and L-valine biosynthesis (Fig. 7). These pathways contribute to various cellular processes and metabolic activities within the microbial communities.

In order to gain a better understanding of how the prokaryotic communities in the Shulgan-Tash cave have adapted to the low-nutrient conditions, we examined their metabolic capabilities in CO_2_ fixation, methane metabolism, nitrogen metabolism, and sulfur metabolism. Carbon metabolism in the microbial communities of the cave involves several pathways, including the TCA cycle, pentose phosphate pathway, glycolysis, gluconeogenesis, and the Calvin-Benson-Bassham cycle (CBB). Additionally, the reductive TCA cycle (rTCA cycle) and reductive acetyl CoA cycle (Acetyl CoA) can be utilized for autotrophic carbon assimilation. Among the biofilms, the abundance of the CBB cycle was higher in “Azurite”, “Coralline”, and “WhiteRhizoidThin” biofilms, while the rTCA cycle or acetyl CoA cycle were more abundant in “CaveCurd”, “Gray2Floor”, “WhiteWavyEdge”, and “WhiteRhizoidFlat” biofilms.

Methanogenesis was predicted in all biofilms of the cave. Methanogenesis from acetate (METH-ACETATE-PWY) was more specific to “CaveCurd” biofilms, whereas methanogenesis from H_2_ and CO_2_ (METHANOGENESIS-PWY) was more specific to “WhiteWavyEdge” and “WhiteRhizoidFlat” biofilms.

Three nitrogen metabolism pathways were identified in the microbiomes of the cave: denitrification (DENITRIFICATION-PWY), assimilatory nitrate reduction (PWY-5675), and nitrifier denitrification (PWY-7084).

Sulfur-related metabolic pathways were also predicted in the cave biofilms. These include the superpathways of S-adenosyl-L-methionine and L-methionine biosynthesis (by sulfhydrylation and transsulfuration), sulfate assimilation and cysteine biosynthesis, as well as the superpathway of sulfur oxidation and sulfate reduction (assimilatory). The proportion of sulfate reduction (assimilatory) enzymes was highest in “Coralline’’ biofilms, while “CaveCurd” and “Gray2Floor” biofilms had a higher abundance of enzymes involved in sulfate assimilation and cysteine biosynthesis.

### Participation in the dissolution and precipitation of calcium carbonate

**Fig. 8:**
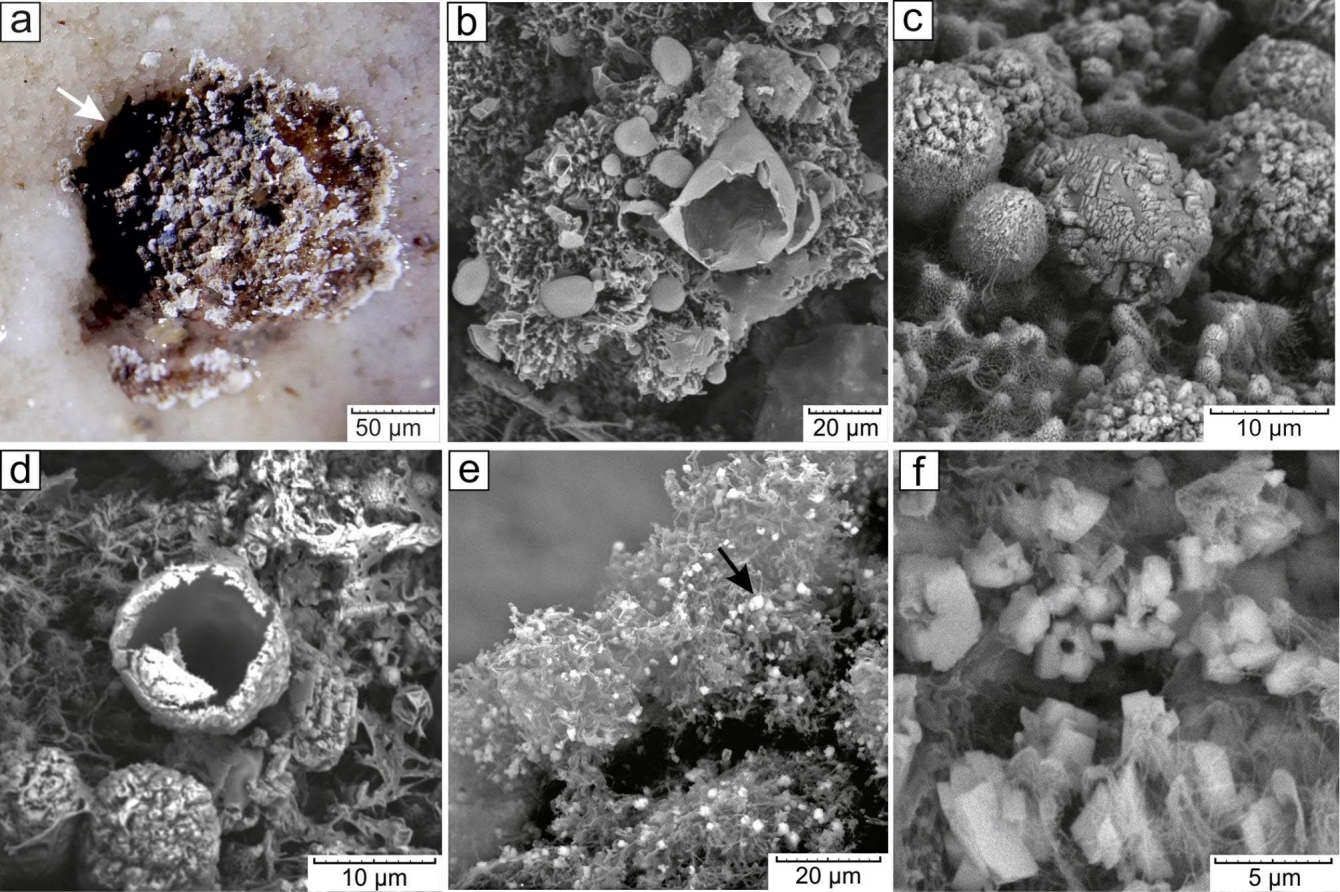
Scanning electron microscopy of calcified biofilms: (**a**) etching pits on calcite crusts under the colonies in “BrownWhite” biofilm; (**b**) partial substitution of EPS structures with CaCO_3_ in a “BrownWhite” biofilm colony; (**c**) CaCO_3_ globules in a “BrownWhite” biofilm colony; (**d**) hollow structure of a CaCO_3_ spheroid; (**e**) Calcite microaggregates on the aerial mycelium of a “WhiteWavyEdge” biofilm colony; (**f**) сalcite microaggregates in a “CaveCurd” biofilm colony, residual channel in the center of colony marked with the arrow

The dissolution of the carbonaceous substrate beneath the growing colonies was observed in several biofilms. This phenomenon was most pronounced in the “BrownWhite” biofilms, where the depth of etching pits on the colonized speleothemes occasionally reached 0.5-1 mm (Fig. 8a). On the other hand, secondary carbonate mineralization was present in all types of biofilms, with a particularly notable occurrence in biofilms dominated by *Actinobacteria*.

Azurite biofilms formed bubbly structures with a high content of CaCO_3_ (Fig. 8b). Formation of CaCO_3_ globules during mineralization was characteristic of 2d-types colonies with a reticulo-nodular structure and spheroid inclusions (Structure no. 2: see Sect. 3.2). We suggest that this colony shape represents spheroid elements pseudomorphs (Fig. 3f). Microbialites in the form of CaCO_3_ globules were 10–12 μm in size (Fig. 8c), sometimes with a hollow internal structure traced on the chips (Fig. 8d).

The “WhiteWavyEdge” biofilm exhibited a different type of microbialite, small aggregates (2–5 μm) of calcium carbonate that formed on aerial mycelium. Similar formations were occasionally found in “Coralline”, “WhiteRhizoidFlat”, and “CaveCurd” biofilms (Fig. 8e). These aggregates consisted of rhombohedral crystals characteristic of calcite. Often, a central channel could be observed within the aggregate, likely resulting from the microbial filament around which the colonies of microbial cells grew (Fig. 8f).

## Discussion

### The Shulgan-Tash cave exhibits a unique abundance and diversity of microbial fouling

Between approximately 60,000 and 14,000 years ago, permafrost existed in the area of the Shulgan-Tash Cave. The development of biofilms under Pleistocene permafrost conditions seems highly unlikely as they require positive temperatures and the presence of liquid water. The necessary conditions for biofilm formation did not emerge until approximately 14,000 years ago when the permafrost degraded, positive temperatures were established within the cave interior, and its connection with external ecosystems was restored [10].

The combination of ecological factors in the Shulgan-Tash cave appears to have influenced the formation of various biofilm morphotypes. We have identified 12 morphotypes that differ in structure, shape and color (Figs. 2 and 3). Similar biofilms have been described in underground cavities in rocks of various lithologies, including carbonates, basalts, phyllite schists, and sandstones [18, 19, 21, 23–25]. In the Shulgan-Tash cave, the most abundant development of colored biofilms is observed between 110 and 300 m from the entrance, where the active air exchange with the surface occurs (Additional file 1: Fig. S2, 4). The mixing of air currents with varying moisture content leads to substantial condensation on the cave walls. This condensation process contributes to the granular disintegration (“micritization”) of the walls and the accumulation of insoluble eluvial sludge on their surfaces. The loosened substrate, enriched with phyllosilicates, provides favorable conditions for the development of *Actinobacteria*. Furthermore, condensation processes play a crucial role in aerosol deposition within the cave air, including the transport of microorganisms from the day surface [72], which contributes to the microbial diversity within the cave. In contrast, the biofilms found in the farthest parts of the cave are not colored and differ from the “cave silver” type. Previous literature primarily described biofilms with gray, white, and yellow shades [23, 24], and the occurrence of the colored biofilms close to the entrance, transitioning to the white biofilms in the distant parts of the cave, aligns with these findings.

One indicator of anthropogenic impact is the prevalence of *Proteobacteria* in the biofilms. The dominance of *Proteobacteria* can be attributed to the introduction of organic matter into these areas by humans [7]. For instance, in the Lascaux cave in France, famous for its rock carvings, that has experienced significant anthropogenic impact, the microbial community consisted almost entirely of *Proteobacteria* [73]. The presence of a significant number of bacteria from the *Bacteroidia* class in cave microbiomes is also considered a bioindicator of anthropogenic influence [8]. Recent studies have shown that the proportion of *Bacteroidetes* in the Lascaux cave was 6.0% compared to 0.1% in other caves from the same region unaffected by human activity, while *Euryarchaeota* and *Woesearchaeota* were not found there [72]. In the microbial films of Shulgan-Tash, a small proportion of *Bacteroidetes* was found in only one type of microbial film (< 2%, ranging from 0.11% in “WhiteRhizoidDense” to 1.9% in “BrownWhite”), indicating a limited anthropogenic impact on the speleological site.

The low representation of the anthropogenic component of the studied communities can hardly be explained by the low attendance of the Shulgan-Tash cave, since this place was very popular among tourists and researchers in the second half of the XXth century before the access was limited. It can be assumed that an ecosystem quite resistant to the intruders has developed in the cave, which was facilitated not only by specific conditions, but also by the production of antibiotics and secondary metabolites by some microorganisms preventing the spread of invasive microbiota. The special attention should be given to the low representation of fungi in further studies.

All biofilms of the cave, except for the “CaveCurd” morphotype, were dominated by representatives of *Actinobacteria*, especially those of the genus *Crossiella* (Fig. 4a, c). The prevalence of *Actinobacteria*, specifically the genera *Crossiella, Euzebya*, and *Pseudonocardia*, has also been observed in other studies of European karst caves, such as Altamira, Sloup-Šošůvka, Pajsarjeva jama, Santimamiñe, Doña Trinidad, Sorcerer’s, as well as volcanic caves in Hawaii and New Guinea [15, 19, 21, 24, 74–77]. *Actinobacteria* exhibit a wide range of morphologies associated with the shape and color of their mycelium, as well as the structure and appearance of their spores [78]. The structural elements of the microbial films in the Shulgan-Tash cave displayed various filaments with branched and tangled loops, as well as spherical (orbed) structures consisting of conglomerates of small coccoid cells or individual large spheres (Fig. 3), which correspond to the morphologies of *Actinobacteria* [79].

The branched and tangled loop 3d- and 2d-morphologies, as well as the presence of large spheres, are common types of microbial filaments in the microbial film communities of underground environments [20–22, 80]. These microbial films are primarily dominated by members of the family *Pseudonocardiaceae*, which exhibit a wide variety of morphological elements, such as branching and looping filaments (Fig. 3a, c, f), individual sporangia carrying spore chains, sporangia bundles with single or chain-like spores (Fig. 3c), and the formation of rounded sporangia containing spores or pseudosporangia-like structures (Fig. 3f) (structures resembling sporangia or sclerotia) (https://atlas.actino.jp/).

Certain *Actinobacteria* are capable of producing diffusible pigments, which can vary in color depending on the strain and age of the culture, ranging from red, yellow, orange, pink, and brownish to distinctly brown, greenish brown, blue, or black [81]. *Streptomyces*, for example, can exhibit a range of colors depending on the strain [82]. Some members of *Pseudonocardia* can form creamy white aerial mycelium and orange-yellow to yellow-brown substrate mycelium [83]. We speculate that the coloration of the biofilms in the cave is associated with the prevalence of *Actinobacteria*. However, we did not manage to find a convincing connection between the taxonomic composition of biofilms and their coloration (Additional file 1: Fig. S15).

*Acidobacteria* were found in all biofilms of the Shulgan-Tash cave and were represented by *Blastocatellia*_11–24 (formerly subgroup Gp4), *Pyrinomonadaceae*_RB41 (formerly subgroup Gp4), and unclassified *Vicinamibacteria* subgroup 17 (formerly *Acidobacteria* subgroup Gp17). The acidic environment provides an advantage for the growth of *Acidobacteria* and serves as one of the predictors for their presence in bacterial communities [84]. However, representatives of this phylum are widely distributed in various habitats, including soils and subsurface sediments [85]. *Acidobacteria* used to be grouped into 26 separate clades or “subgroups.“ Subgroups Gp4, Gp6, Gp7, and Gp16 are associated with alkaline conditions (pH ~ 8.5) [85]. Representatives of *Acidobacteria* subgroup Gp4 are frequently found in epilithic biofilms, water bacterial mats, and make a significant contribution to the microbial community structure in caves [17, 86–92]. Therefore, it is not surprising that they were present in the Shulgan-Tash cave. *Blastocatellia* 11–24 (ASV7 and ASV10) was found to show high similarity with the corresponding sequences of some non-cultivated species found in various caves (Additional file 1: Fig. S13). Despite their low abundances, *Acidobacteria* play an important role in fouling propensity [93]. Overall, *Acidobacteria* are well-suited for survival in oligotrophic niches, but their specific role in the biogeochemical cycle remains unknown.

Bacteria belonging to the class *Alphaproteobacteria* were primarily found on pure carbonate substrates in the remote areas of the cave. It is known that the genera *Pedomicrobium* and *Hyphomicrobium* (class *Alphaproteobacteria*) found in the Shulgan-Tash cave are capable of facultatively oxidizing iron and manganese and are often associated with ferromanganese deposits in cave ecosystems [6, 94]. This raises concerns about their potential impact on the Paleolithic paintings in the Shulgan-Tash cave, as the development of Fe-Mn mineralization has been observed on some drawings.

Representatives of the order *Rhizobiales* are mostly known as a group of soil bacteria that induce nodule organogenesis in legume roots and fix atmospheric nitrogen for plant growth [95]. However, there is evidence of their presence in oligotrophic cave systems unrelated to plant rhizospheres [6].

Certain members of the phylum *Chloroflexi* were present among the dominant taxa in the “Gray2Floor” and “WhiteRhizoidFlat” biofilms, but they could only be reliably identified at the phylum level (Additional file 1: Fig. S10). The phylum *Chloroflexi* is a diverse and broad group, with many of its microorganisms being uncultivable, which makes it challenging to assess their metabolic functions [96]. The common occurrence of *Chloroflexi* in oligotrophic environments, including caves, suggests their pre-adaptation to thrive under limited nutrient conditions [23, 97].

Bacteria belonging to the phylum *Planctomycetes* were significantly represented (4.35%) in the “CaveCurd” biofilm. The sequences corresponding to the phylum *Planctomycetes* were assigned to the genera *Gemmata*, *Blastopirellula*, and *Pirellula*, respectively (Additional file 1: Fig. S7, 10). Members of this group are, likely, chemoheterotrophs [98–100].

The species of *Deltaproteobacteria* found in the studied samples mostly belonged to the order *Myxococcales*. These microorganisms prefer well-moistened substrates. The prevalence of myxobacteria in the areas of caves with an active supply of karst waters has also been reported in previous studies [101].

The cyanobacteria were represented by the recently described family *Obscuribacteraceae*, which is found in the dark and is characterized by a complete absence of a photosynthetic apparatus [102].

### “CaveCurd” biofilm has a different taxonomic composition

In the “CaveCurd” biofilm, the dominant taxon was an uncultured bacterium belonging to the order Ga0077536 (*Gammaproteobacteria*) (~ 19%). The genome of this bacterium was assembled from a metagenomic sample obtained from biologically active filters at the drinking water treatment plant [103] and its functional analysis was carried out [104]. This taxon has been also found associated with marine organisms such as corals [105], the planktonic bacterial community associated with spring phytoplankton blooms [106], and prokaryotic communities from a lava tube cave [104]. The uncultured bacterium from the gammaproteobacterial order Ga0077536 dominated in the moonmilk community [104]. In contrast, *Actinobacteria* accounted for only ~ 2% of the total phyla (Fig. 4). This distribution is fundamentally different from other samples from the Shulgan-Tash cave. The differences can be explained by the fact that the “CaveCurd” biofilm developed in places located deep in the cave (Fig. 1; Additional file 2), where *Gammaproteobacteria* distribution was limited to abundantly moistened substrates (Fig. 6) characterized by higher concentrations of CO_2_ (Additional file 1: Fig. S3). The predominant clumps of small cocci with a rough surface in the “CaveCurd” colonies were covered by an EPS-like matrix (Fig. 3g, h), similar to microbial films where uncultured representatives of *Gammaproteobacteria* have been detected [23, 107, 108]. Previous analysis of the Ga0077536 genome has shown that it plays a relevant role in the methane cycle, as evidenced by the presence of genes likely involved in sulfur, nitrogen, and carbon metabolism [104]. This finding is also consistent with our PICRUSt2 analysis data (Fig. 7). Thus, the most distant part of the Shulgan-Tash Cave is colonized by a metabolically diverse prokaryotic community.

### Influence of environmental factors on the taxonomic distribution of microbial communities

Analysis of environmental factors revealed that the presence of *Actinobacteria* was primarily observed in the proximal parts of the cave, which have a significant climatic connection with the surface (Additional file 1: Fig. S2). Additionally, *Actinobacteria* showed a preference for substrates rich in phyllosilicates and biogenic elements such as phosphorus (P) and sulfur (S) (Fig. 6). Clay minerals, known for their high capacity to adsorb macromolecules, can accumulate organic substances, creating favorable conditions for the growth of *Actinobacteria* [109]. A similar pattern of *Actinobacteria* development in the areas close to the cave entrance, followed by a decrease and succession by *Proteobacteria* in the distant parts, has been observed in other cave systems as well [110]. The confinement of *Actinobacteria* to the proximal zone of the Shulgan-Tash cave may be attributed to specific geological microprocesses. The flow of warm air in summer, followed by moisture condensation, provides a source of water and leads to corrosion, erosion, and granulation of limestone, resulting in the accumulation of eluvial material containing phyllosilicates on the cave walls. This substrate provides favorable conditions for actinobacterial colonization.

Caves and other subterranean habitats are limited in food resources due to the absence of photosynthesis [111–113]. Seeping water and dissolved organic carbon (DOC) entering the cave from water movement through the unsaturated zone and underlying groundwater system are often the only sources of organic carbon in caves. DOC is the primary source of organic carbon in karst rocks, with its concentration likely decreasing with depth [112, 114]. DOC consists of a complex mixture of organic molecules, such as polysaccharides, lignin, humic acids, and fulvic acids, which vary in their quality as energy sources. In this context, a model of ecological succession associated with microbial community dynamics can be assumed as processing of DOC, as observed in soils [115]. As the pool of organic carbon becomes increasingly oxidized and nutrient-poor, bacteria capable of utilizing such substrates gain a selective advantage. Differential abundance analysis revealed taxonomic differences between the floors of the Shulgan-Tash cave (Fig. 5c), which may be attributed to variations in the availability and oxidation state of the organic matter. In the future, we plan to conduct a more detailed analysis of the organic matter in the Shulgan-Tash cave. Representatives of the *Planctomycetota* and *Acidobacteria* phyla are well represented on both cave floors. *Acidobacteria* are capable of growing in sites where carbon has been highly oxidized [115]. *Planctomycetota* is well-adapted to the extremely oligotrophic niche within caves and can grow on poorly reduced substrates [116]. It is possible that the bacterial community in the biofilms specializes in the stepwise catabolism of available allochthonous DOC. Additionally, *Actinobacteria*, *Proteobacteria*, and *Acidobacteria*, which are characteristic of cave microbiomes, may have adapted to oligotrophic conditions through chemoautotrophic pathways of energy metabolism [113]. Therefore, a more comprehensive investigation of energy metabolism of the cave community in relation to cave geochemistry is needed in the future.

### Functional reconstruction of the cave microbiome metabolic pathways

Since the majority of microorganisms in natural habitats remain uncultivated, a metagenomic approach based on high-throughput sequencing is crucial for studying cave microbiomes. For a detailed analysis of natural microbiomes, shotgun sequencing provides optimal results. However, preliminary studies often employ the 16S rRNA gene metabarcoding to classify samples according to characteristic communities in an ecosystem [117]. Recently, several tools, including PICRUSt2, Tax4Fun2, Piphillin, Faprotax, and Paprica have been developed for the functional prediction of microbiome metabolism based on 16S rRNA gene sequence data. These tools enable the prediction of metabolic potential of microbial communities from diverse habitats [118] before conducting metagenomic shotgun sequencing. The main challenge faced by microbial communities in carbonate caves is the oligotrophic habitat conditions [119, 120]. It is generally assumed that carbonate cave communities are supported by allochthonous carbon supplied from the surface [121, 122]. These caves are characterized by low nutrient availability and high calcium content. It is likely that these communities are specially adapted to survive in such conditions, potentially supported, at least in part, by a strategy of primary production based on inorganic nitrogen [119]. To understand how the prokaryotic community in the Shulgan-Tash cave is adapted to low-nutrient conditions, we focused on CO_2_ fixation, methane, nitrogen, and sulfur metabolism.

Our data (Fig. 6) also indicate that *Actinobacteria* in the cave are associated with the presence of Al_2_O_3_, P_2_O_5_, and S, which suggest substrates rich in phyllosilicates and biogenic elements. However, there is evidence suggesting that cave microbial communities may not rely solely on external sources of organic carbon inputs from the surface. Selensky et al. demonstrated that biofilm bacteria in lava caves actively fix inorganic carbon despite the availability of organic carbon [123]. Additionally, the utilization of carbon substrates by cave microbial communities was found to be lower than that of bacteria outside the cave, and methane-based chemoautotrophic pathways may play a significant role in primary production [113]. In fact, our predictions of metabolic pathways in the Shulgan-Tash cave microbiomes indicate a high potential for CO_2_ fixation. Bacteria of the genus *Nitrosospira* can carry out CO_2_ fixation through the CBB cycle [124]. Martin-Pozas and coauthors [125] associated CO_2_ assimilation in cave sediments covered by moonmilk deposits with *Crossiella* and *Nitrosococcaceae* wb1-P19, which were dominant in the Shulgan-Tash biofilms (Fig. 4c). The predominant sulfur-oxidizing *Gammaproteobacteria* in the biofilms are capable of performing the reductive TCA cycle, which is rather rare in nature [126]. Oligocarbophiles of the genus *Hyphomicrobium*, which also require CO_2_, were documented in the biofilms from the deep parts of the cave. They can grow under anaerobic conditions relying on denitrification or mixed acid fermentation. Some representatives of this genus possess genes involved in N_2_ fixation [127]. Although the nitrogen fixation pathway was not predicted for the biofilms in the Shulgan-Tash cave, other subsystems related to nitrogen metabolism, including nitrification (e.g., ammonia monooxygenase, hydroxylamine dehydrogenase), denitrification (e.g., nitrate reductase, nitrite reductase (NO-forming), periplasmic nitrate reductase), assimilatory and dissimilatory nitrate reduction (e.g., assimilatory nitrate reductase, nitrite reductase (NADH), ferredoxin-nitrite reductase), were expected to be present in the studied Shulgan-Tash cave community.

During carbon assimilation via the CBB cycle, nitrogen chemolithotrophy is a characteristic process in which ammonia or nitrite acts as an electron donor. In the Shulgan-Tash biofilms, the ability to oxidize ammonia (NH_3_) to nitrite (NO_2_) may be attributed to the presence of ammonia-oxidizing archaea, such as *Nitrosopumilus* and *Nitrososphaera* (Additional file 1: Fig. S8, 9). Further oxidation to nitrate may be facilitated by nitrite-oxidizing bacteria, such as *Nitrospira*, *Nitrosococcacea* wb1-P19 and *Nitrosococcus*. *Nitrosococcacea* wb1-P19 is the most abundant autotrophic nitrite-oxidizing bacterium in the microbial community of the Shulgan-Tash (Fig. 4c), and it appears to contribute to primary organic production.

The involvement of the cave microbiome in nitrogen oxidation is indirectly supported by the composition of drip waters. Higher concentrations of NO_2_ and NO_3_ were observed in water from cavities where the walls and ceilings were covered with “cave silver” microbial films, compared to water from distant cavities without visible microbial fouling (Additional file: Table S2, 3). Similar elevated levels of nitrogen compounds have been observed in groundwater of other caves with biofilms [128, 129]. If our assumption is correct, autotrophic CO_2_ fixation would be one of the main sources of carbon input, as nitrification is always accompanied by this process. The main question then arises regarding the source of ammonia or ammonium salts in the cave. Since there is no permanent population of bats in the Shulgan-Tash cave, these compounds may be provided by nitrogen-fixing activity of certain members of the microbial community that have yet to be identified, or they may enter from the surface through seepage waters. However, the latter seems less likely since readily available nitrogen compounds would be utilized by soil bacteria. Chemical analyses of filtered water samples from various cave cavities without fouling did not reveal any reduced nitrogen compounds. The production of ammonia can be associated with the heterotrophic activity of *Crossiella*, which is widely represented in the Shulgan-Tash cave.

Recently, it has been shown in situ that cave communities can efficiently consume methane [125]. The involvement of methanogens in the methane cycle in karst caves is still poorly understood. It was demonstrated [130] that although methanogens were present in the cave community, methane production by them was not detected, while methane-oxidizing bacteria actively participated in methane consumption. Therefore, the role of methane in the cave ecosystem needs to be addressed. In any case, the microbial communities of the Shulgan-Tash cave exhibit a wide range of strategies for acquiring essential elements. However, a comprehensive assessment of the energy balance in these biotopes is needed.

### Microbially induced mineral formation in the Shulgan-Tash cave

Biofilms that develop on the carbonate substrates can have both destructive (local corrosion of calcite) and constructive (microbially mediated precipitation of calcium carbonate) effects. The “BrownWhite” morphotype showed the most pronounced dissolution of calcium carbonate, accompanied by the formation of etching pits under the growing biofilm.

There are active and passive mechanisms involved in calcium carbonate mineralization. Active precipitation of carbonates by bacteria is associated with ion exchange processes across the cell membrane through ion pumps or calcium and/or magnesium channels [131]. It is believed that the precipitation of calcite by heterotrophs in calcium-rich environments is a result of bacterial cells detoxifying excessive Ca^2+^ [132]. CO_3_^2–^ and HCO_3_^–^ ions are generated through the absorption of CO_2_ from the atmosphere by bacteria, facilitated by the carboanhydrase enzyme [133, 134].

Passive participation in mineral formation, known as biologically determined mineralization, occurs through the modification of the microenvironment’s chemical composition by bacterial metabolites. This leads to changes in pH, Eh, pCO_2_, and the presence of CO_3_^2–^ and HCO_3_^–^ ions [134]. Changes in the media chemistry that induce carbonate precipitation can be observed during processes such as ammonification in the nitrogen cycle [131, 134], anaerobic sulfate reduction [135], and photosynthesis [136]. Studies have also demonstrated the importance of bacterial extracellular polymeric substances (EPS) in microbially induced mineral formation [137]. It is suggested that negatively charged EPS macromolecules can serve as acceptors for Ca^2+^ and Mg^2+^ cations [138, 139].

Precipitation of calcium carbonate was observed in all biofilm morphotypes of the Shulgan-Tash cave, and the localization of the process consistently corresponded to the presence of microbial filaments characteristic of *Actinobacteria* (Fig. 8). *Actinobacteria* are known to be involved in carbonate mineral formation. For instance, 61% of *Actinobacteria* strains isolated from the Altamira Cave produced calcite and vaterite crystals in nutrient media [140]. Similarly, bacteria of the genus *Streptomyces*, isolated from the moonmilk of the Grotte des Collemboles cave in Belgium, exhibited the ability to form carbonate minerals through processes such as the nitrogen cycle and CO_2_ absorption by carboanhydrase [28]. The representatives of the genus *Crossiella* have been supposed to induce calcite deposition on certain historical structures in China [141]. Representatives of this genus are abundantly present in the biofilms of the Shulgan-Tash cave, suggesting their active involvement in recalcification.

Carbonate spheroids in actinobacterial colonies with similar sizes and morphology have been described in the Altamira Cave [20], Tomba della Scimmia and Tomba del Colle in Italy [109, 142], in colored microbial mats from the Azores, Canaries, and Hawaiian lava caves [22]. In a number of these objects, similar calcite microaggregates (the so-called «nest-like aggregates») were found on aerial mycelium [20]. Despite that in the Shulgan-Tash cave the infiltration waters are oversaturated with calcite (as speleothems actively grow in the cave), its chemogenic deposition in biofilms seems unlikely, since in most of the cases, a selective mineralization was observed, affecting only individual elements of the microbial mass, such as spheroid structures, aerial mycelium, and EPS. At the same time, dissolution of the carbonate substrate by microbial metabolites is often observed beneath the colony. It suggests the formation of microbialites by an active mechanism, for example, due to detoxification from Ca^2+^ ions, the source of which can be a carbonate substrate. The infiltration waters of the cave contain nitrogen compounds, and some bacteria that are present in the biofilms, such as *Nitrospira*, can participate in ammonification [143]. This provides the prerequisites for passive microbially induced CaCO_3_ mineral formation.

Indeed, representatives of microbial biofilms play a crucial role in the dissolution and precipitation of carbonates, posing a potential threat to the preservation of Palaeolithic paintings. However, one cannot disregard the influence of biologically active compounds that form during microbial activity. These compounds, including organic acids and enzymes, might initiate chemical reactions upon contact with mineral substrates, leading to erosion or alteration of the cave walls. Furthermore, microbial EPS can bind to mineral surfaces, facilitating the adhesion of microbial communities to cave walls and trapping mineral particles, thereby impacting the cave surface.

Hence, the observed biocorrosion of cave surfaces could stem from various metabolic processes likely executed by cave biofilms. These intricate interactions emphasize the importance of understanding microbial activities in caves, especially concerning their potential effects on the preservation of valuable cultural and natural heritage sites.

## Conclusion

The Shulgan-Tash cave serves as an intriguing example of an oligotrophic ecosystem with an unexpectedly abundant and diverse microbiota. Despite the abundance of fouling morphotypes identified by external features, they are represented by three types of biofilms based on the similarity of their ultrastructure. At the same time, according to the results of molecular taxonomic analysis, two main clusters of microbial communities were identified, grouped according to the nuclear composition of the dominant taxa. The first of them is a variation of the community with the overwhelming dominance of *Actinobacteria*. The specific species composition of these biofilms reflects variations in environmental conditions in different cave zones, primarily the chemical composition of the substrates, especially the content of phosphorus and nitrogen, as well as microclimatic factors such as temperature stability, relative humidity, ventilation, and CO_2_ content. The observed differentiation of the prokaryotic communities of the 1st and 2nd floors can also be explained by the different contribution of communication with the surface through the epikarst (including through seepage waters) and air inflow through the cave entrance.

In the deepest parts of the cave, the most isolated from the surface, a special community “CaveCurd” was found, dominated by *Gammaproteobacteria* (Ga0077536), and representatives of *Planctomycetes*, *Alphaproteobacteria*, *Acidobacteria*. This community is characterized by the greatest diversity and the most autonomous metabolism with a high autotrophic potential, which probably corresponds to specific adaptation to cave conditions.

The oligotrophic environment of the Shulgan-Tash cave significantly contributes to the presence of chemolithotrophic microorganisms in the microbiomes of its remote sections. The cave’s microflora potentially utilizes various mechanisms for energy production, including methanogenesis, nitrification, denitrification, sulfur oxidation, and sulfate reduction. Ammonia-oxidizing archaea and nitrite-oxidizing bacteria are widely represented in the biofilms and play an important role in the nitrogen cycle. The distant part of the Shulgan-Tash cave is colonized by a metabolically diverse prokaryotic community, with the dominant bacterium Ga0077536, which may play a relevant role in the methane cycle. *Crossiella* and *Nitrosococcaceae* wb1-P19 potentially possess complete cycles of carbon dioxide fixation and contribute to the production of organic carbon and remineralization. The intense dissolution and deposition of carbonates resulting from the activities of *Actinobacteria* pose a potential threat to the preservation of Paleolithic paintings. As alteration of the cave wall surface due to condensation corrosion is a critical factor influencing the spread of actinobacterial biofilms, it is crucial to continually monitor the microclimatic conditions in the cave and, if necessary, implement measures to limit air exchange, especially during the summer months.

### Electronic supplementary material

Below is the link to the electronic supplementary material.

Additional file 1. Supplementary data and results. **Fig. S1.** Biofilm sampling at the Arch of the Hall of Paintings. **Fig. S2**. Scheme of air circulation in the Shulgan-Tash Cave. **Fig. S3**. Microclimatic parameters (T, RH, CO_2_) in the Shulgan-Tash cave. **Fig. S4**. Spatial distribution of biofilm morphotypes inside the Shulgan-Tash Cave. **Fig. S5.** Changes in the absolute moisture content in the air at the surface and the potential for water condensation in the cave. **Table S1.** Chemical composition of substrates colonized by the biofilms, according to the results of X-ray fluorescence analysis (mass %). **Fig. S6**. Assessment of the relationship of chemical elements in substrates: (a) correlation matrix (Spearman correlation, results shown significant at *p* < 0.05), (b) DCA – density. **Table S2**. NO_3_ and NO_2_ in drip water, descriptive statistics. **Table S3.** NO_3_ and NO_2_ in drip water, Kruskal-Wallis test / Two-tailed test. **Figure S7**. Relative abundances of the bacterial genus in the communities of the cave biofilms. **Figure S8**. The taxonomic composition of the archaea in the cave biofilms at the genus level. **Figure S9.** The taxonomic composition of the archaea in the cave biofilms at the family level. **Figure S10.** Representation of dominant bacterial taxa in the biofilms of the Shulgan-Tash cave. **Figure S11**. Rooted maximum likelihood RaxML phylogenetic tree based on V4 region of the 16S rRNA genes showing the relationships of *Actinobacteria* members across different sample locations. **Figure S12.** Rooted maximum likelihood RaxML phylogenetic tree based on V4 region of the 16S rRNA genes showing the relationships of the order Ga0077536 members across different sample locations. **Figure S13**. Rooted maximum likelihood RaxML phylogenetic tree based on V4 region of the 16S rRNA genes showing the relationships of *Acidobacteria* members across different sample locations. **Figure S14**. Alpha diversity indices (Chao1 and Inverted Simpson) of bacterial (A) and archaeal (B) communities of Shulgan-Tash cave. **Figure S15**. Heat map showing taxonomic composition of the bacterial communities of cave biofilms at the class level

Additional file 2 Characteristics of the biofilm morphotypes

Additional file 3 Predicted pathway abundance

## Data Availability

The 16S rRNA gene sequencing data have been submitted to the NCBI’s Sequence Read Archive (SRA) under the accession number BioProject PRJNA1018771. bbduk. Available online: https://sourceforge.net/projects/bbmap/. microbiome R package. Available online: http://microbiome.github.io/. Silva 138.1 prokaryotic SSU taxonomic database. Available online: https://zenodo.org/record/4587955/. Digital atlas of *Actinomycetes*: https://atlas.actino.jp/.

## Abbreviations

ASV: Amplicon Sequence Variant
EPS: Extracellular polymeric substances
PCA: Principal Components Analysis
CCA: Canonical Correspondence Analysis
LefSe: Linear discriminant analysis effect size
DOC: Dissolved organic carbon
CBB: Calvin-Benson-Bassham cycle
rTCA cycle: Reductive TCA cycle
