# Supplementary material for: Microbial tapestry of the Shulgan-Tash cave (Southern Ural, Russia): influences of environmental factors on the taxonomic composition of the cave biofilms
Source: Environ Microbiome. 2023 Nov 21;18:82. doi: 10.1186/s40793-023-00538-1 (PMC10662634; doi:10.1186/s40793-023-00538-1)
Supplement: Supplementary file 1 — Additional file 1. Supplementary data and results. Fig. S1. Biofilm sampling at the Arch of the Hall of Paintings. Fig. S2. Scheme of air circulation in the Shulgan-Tash Cave. Fig. S3. Microclimatic parameters (T, RH, CO2) in the Shulgan-Tash cave. Fig. S4. Spatial distribution of biofilm morphotypes inside the Shulgan-Tash Cave. Fig. S5. Changes in the absolute moisture content in the air at the surface and the potential for water condensation in the cave. Table S1. Chemical composition of substrates colonized by the biofilms, according to the results of X-ray fluorescence analysis (mass %). Fig. S6. Assessment of the relationship of chemical elements in substrates: (a) correlation matrix (Spearman correlation, results shown significant at p < 0.05), (b) DCA – density. Table S2. NO3 and NO2 in drip water, descriptive statistics. Table S3. NO3 and NO2 in drip water, Kruskal-Wallis test / Two-tailed test. Figure S7. Relative abundances of the bacterial genus in the communities of the cave biofilms. Figure S8. The taxonomic composition of the archaea in the cave biofilms at the genus level. Figure S9. The taxonomic composition of the archaea in the cave biofilms at the family level. Figure S10. Representation of dominant bacterial taxa in the biofilms of the Shulgan-Tash cave. Figure S11. Rooted maximum likelihood RaxML phylogenetic tree based on V4 region of the 16S rRNA genes showing the relationships of Actinobacteria members across different sample locations. Figure S12. Rooted maximum likelihood RaxML phylogenetic tree based on V4 region of the 16S rRNA genes showing the relationships of the order Ga0077536 members across different sample locations. Figure S13. Rooted maximum likelihood RaxML phylogenetic tree based on V4 region of the 16S rRNA genes showing the relationships of Acidobacteria members across different sample locations. Figure S14. Alpha diversity indices (Chao1 and Inverted Simpson) of bacterial (A) and archaeal (B) communities of Shulgan-Tash cav [file 40793_2023_538_MOESM1_ESM.docx]

**Supplementary materials**


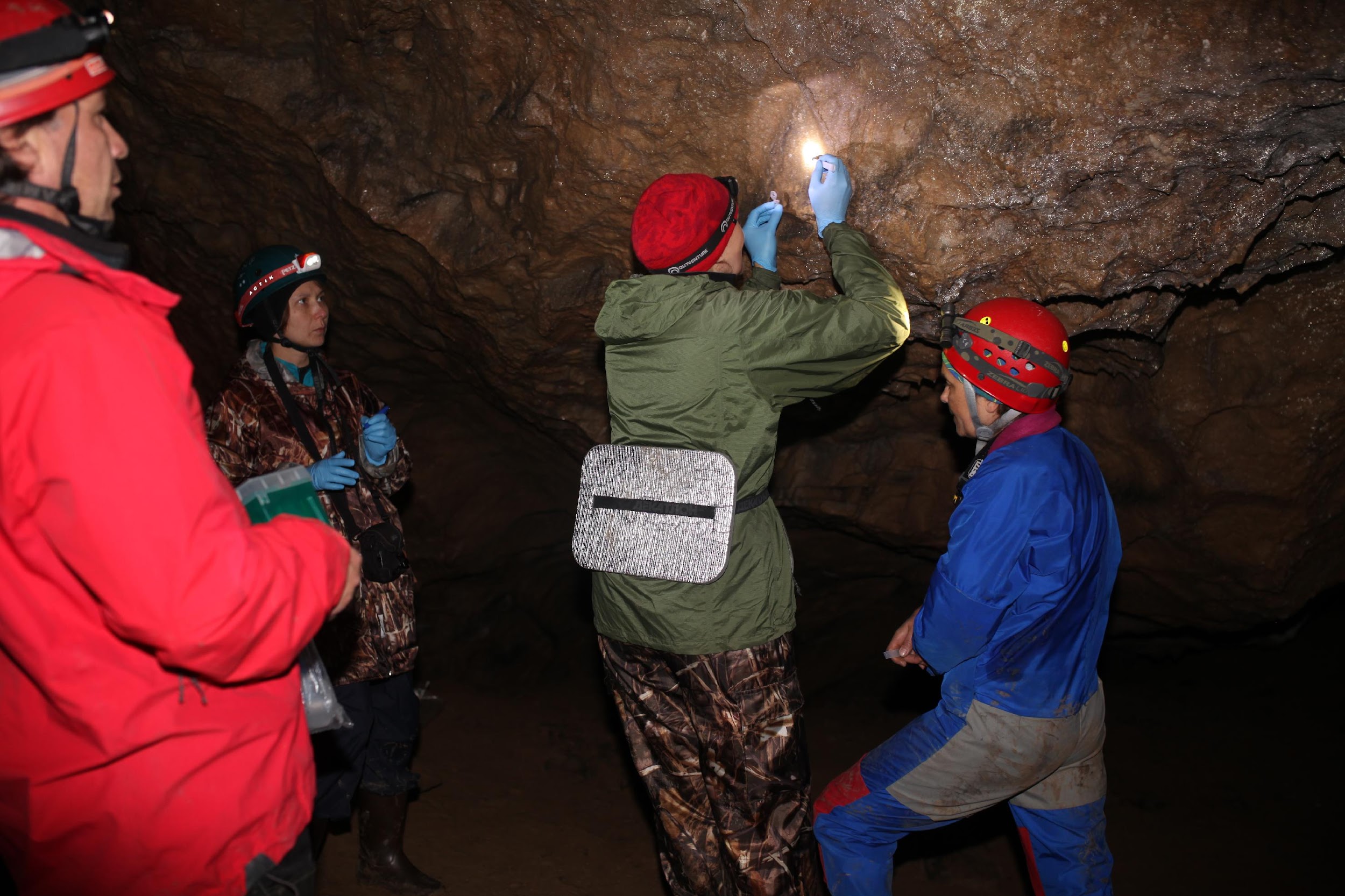


**Fig. S1.** Biofilm sampling at the Arch of the Hall of Paintings.


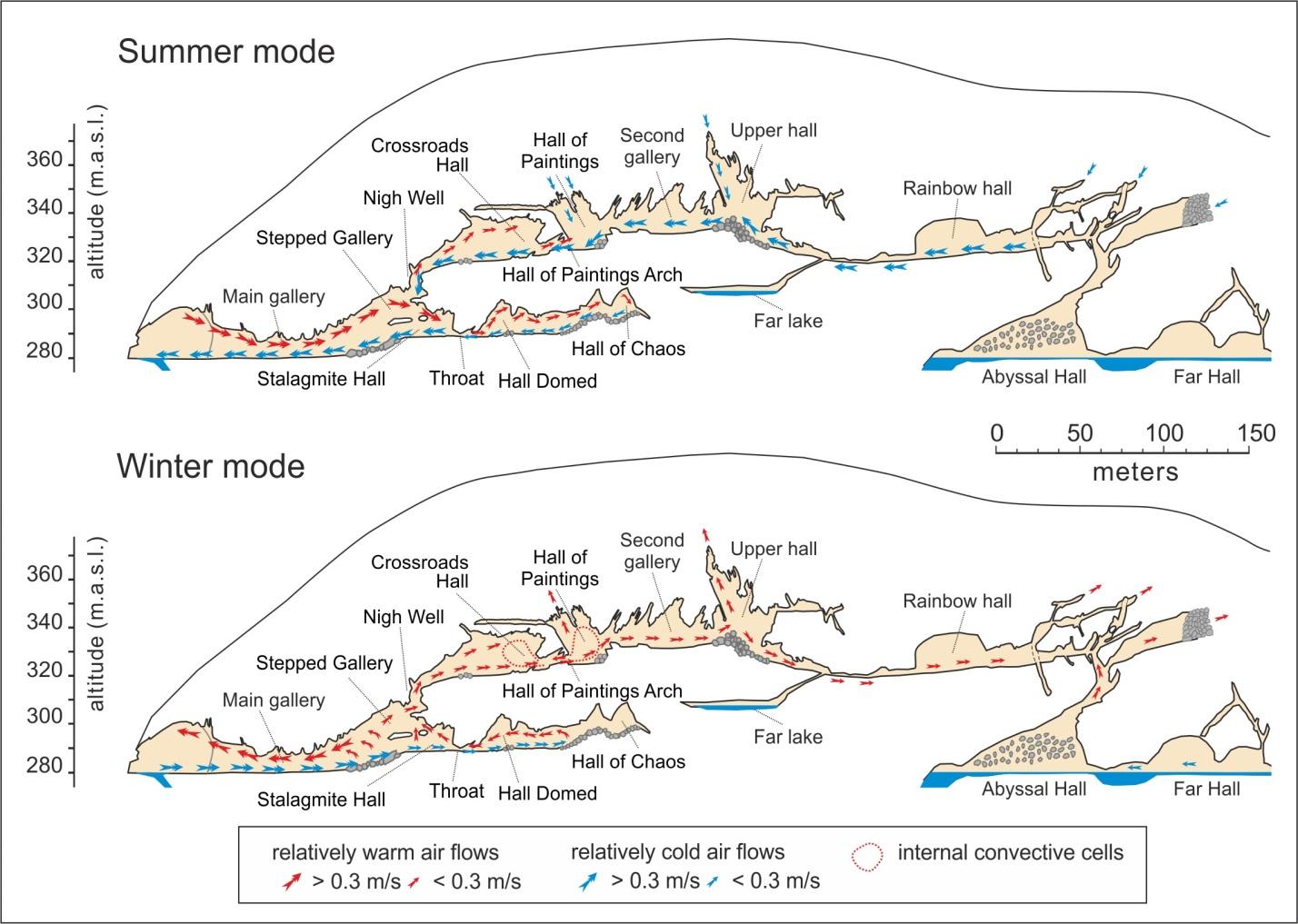


**Fig. S2**. Scheme of air circulation in the Shulgan-Tash cave.


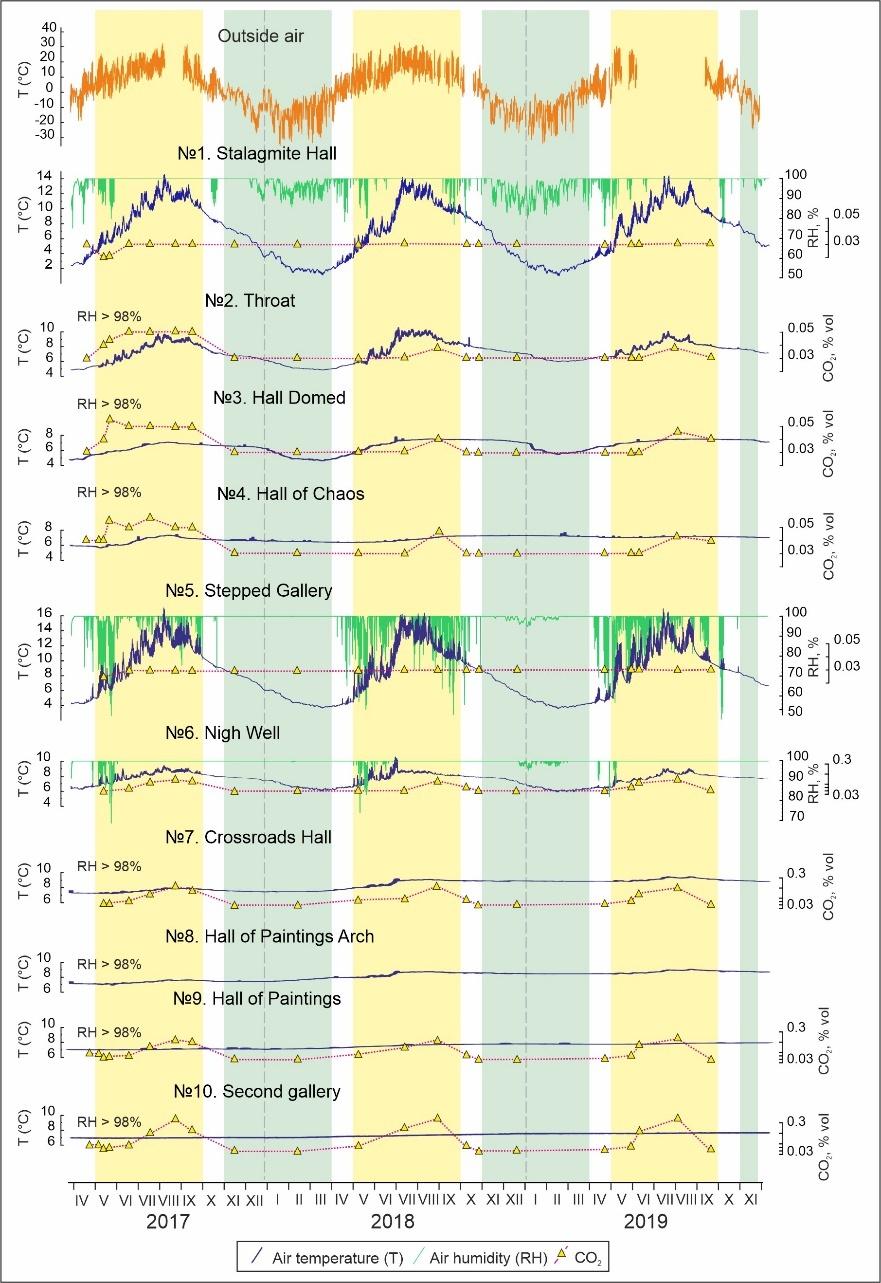


**Fig. S3**. Microclimatic parameters (T, RH, CO_2_) of the Shulgan-Tash cave.


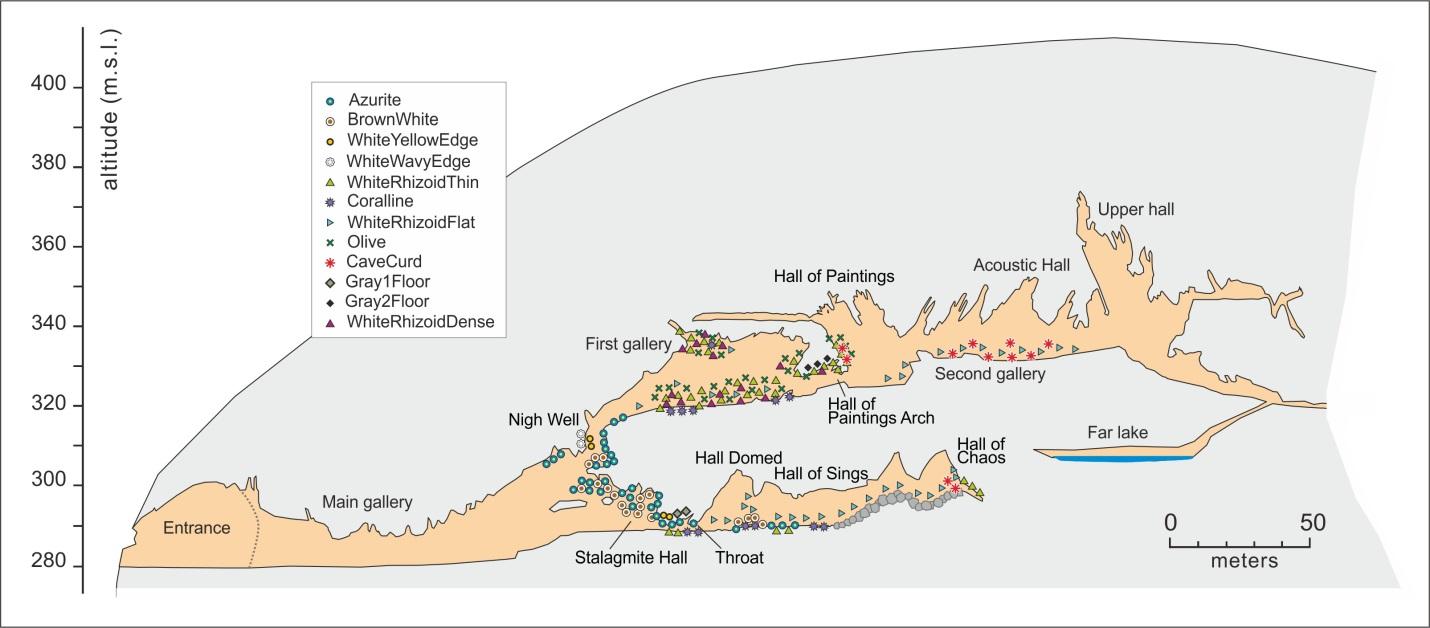


**Fig. S4**. Spatial distribution of biofilm morphotypes inside the Shulgan-Tash cave.


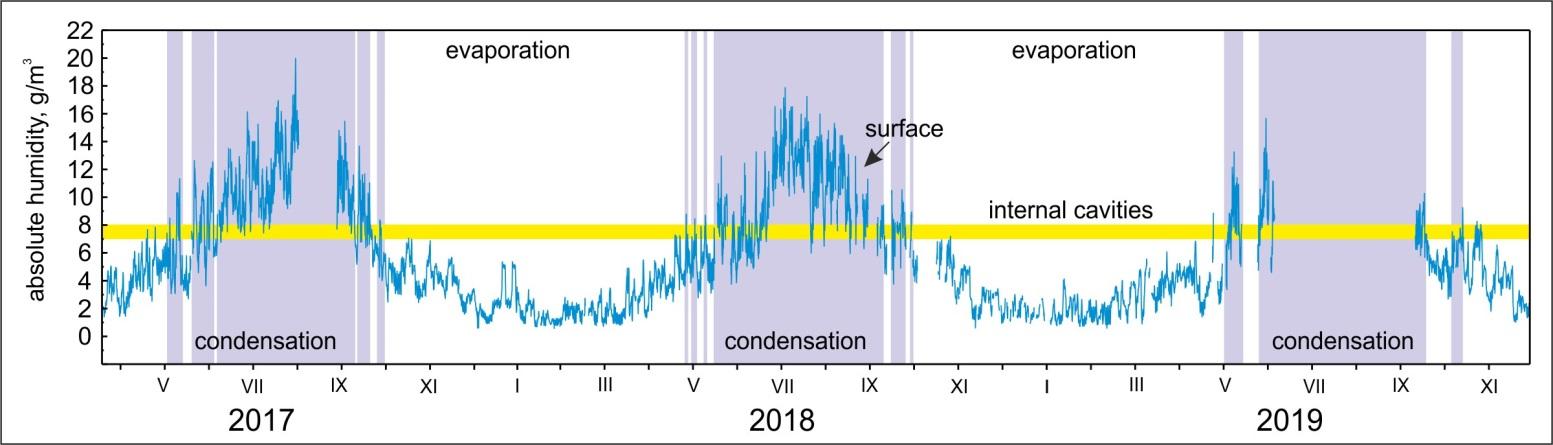


**Fig. S5.** Changes in the absolute moisture content in the air at the surface and the potential for water condensation in the cave.

**Table S1.** Chemical composition of substrates colonized by the biofilms, according to the results of X-ray fluorescence analysis (mass %). Values *in italics* are below the analytical error limit.

| **Biofilm morphotype** | **Substrate*** | **Na_2_O** | **MgO** | **Al_2_O_3_** | **SiO_2_** | **P_2_O_5_** | **S** | **K_2_O** | **CaO** | **TiО_2_** | **MnO** | **Fe_2_O_3_** | **Ignition loss** | **sum** |
| --- | --- | --- | --- | --- | --- | --- | --- | --- | --- | --- | --- | --- | --- | --- |
| Azutite | ST | 0.08 | 0.20 | 0.22 | 0.74 | 0.18 | 0.05 | 0.00 | 54.22 | 0.01 | *0.00* | 0.09 | 44.21 | 100 |
| BrownWhite | ST | 0.06 | 0.26 | 0.63 | 2.67 | 0.12 | 0.04 | 0.06 | 53.00 | 0.04 | *0.01* | 0.17 | 42.94 | 100 |
| WhiteYellowEdge and  Gray1Floor | M | 0.13 | 0.54 | 2.02 | 4.50 | 1.09 | 0.05 | 0.52 | 47.02 | 0.11 | 0.45 | 2.57 | 40.94 | 99.9 |
| WhiteRhizoidThin | LM | 0.08 | 0.34 | 0.23 | 0.53 | 0.16 | 0.02 | 0.00 | 54.87 | 0.01 | 0.03 | 0.23 | 43.45 | 100 |
| Olive | ST | 0.09 | 0.28 | 0.85 | 2.00 | 0.10 | 0.02 | 0.06 | 53.02 | 0.05 | *0.00* | 0.19 | 43.43 | 100.1 |
| WhiteRhizoidFlat | LM | 0.09 | 0.25 | 0.25 | 0.29 | 0.05 | 0.01 | 0.00 | 55.15 | 0.00 | *0.02* | 0.07 | 43.80 | 100 |
| Coralline | ST+ Cl | 0.51 | 1.04 | 6.12 | 38.50 | 0.17 | 0.04 | 0.88 | 27.90 | 0.58 | 0.08 | 2.57 | 21.54 | 100 |
| WhiteWavyEdge | ST | 0.16 | 0.43 | 1.26 | 3.49 | 0.63 | 0.17 | 0.18 | 50.70 | 0.07 | *0.01* | 0.31 | 42.46 | 99.8 |
| WhiteRhizoidDense | ST | 0.09 | 0.23 | 0.42 | 1.33 | 0.06 | 0.01 | 0.00 | 54.27 | 0.02 | *0.00* | 0.10 | 43.36 | 99.9 |
| Gray2Floor | M | 0.09 | 0.45 | 1.08 | 2.61 | 0.18 | 0.03 | 0.16 | 52.15 | 0.05 | 0.04 | 0.26 | 42.87 | 100 |
| CaveCurd | LM | 0.08 | 0.38 | 0.20 | 0.27 | 0.09 | 0.01 | 0.00 | 55.18 | 0.01 | *0.01* | 0.07 | 43.61 | 99.8 |

* ST – calcite speleothems, M – altered (micritized) limestone, ST+ Cl – speleothems with clay interlayers, LM – unaltered limestone, Cl – clay deposits


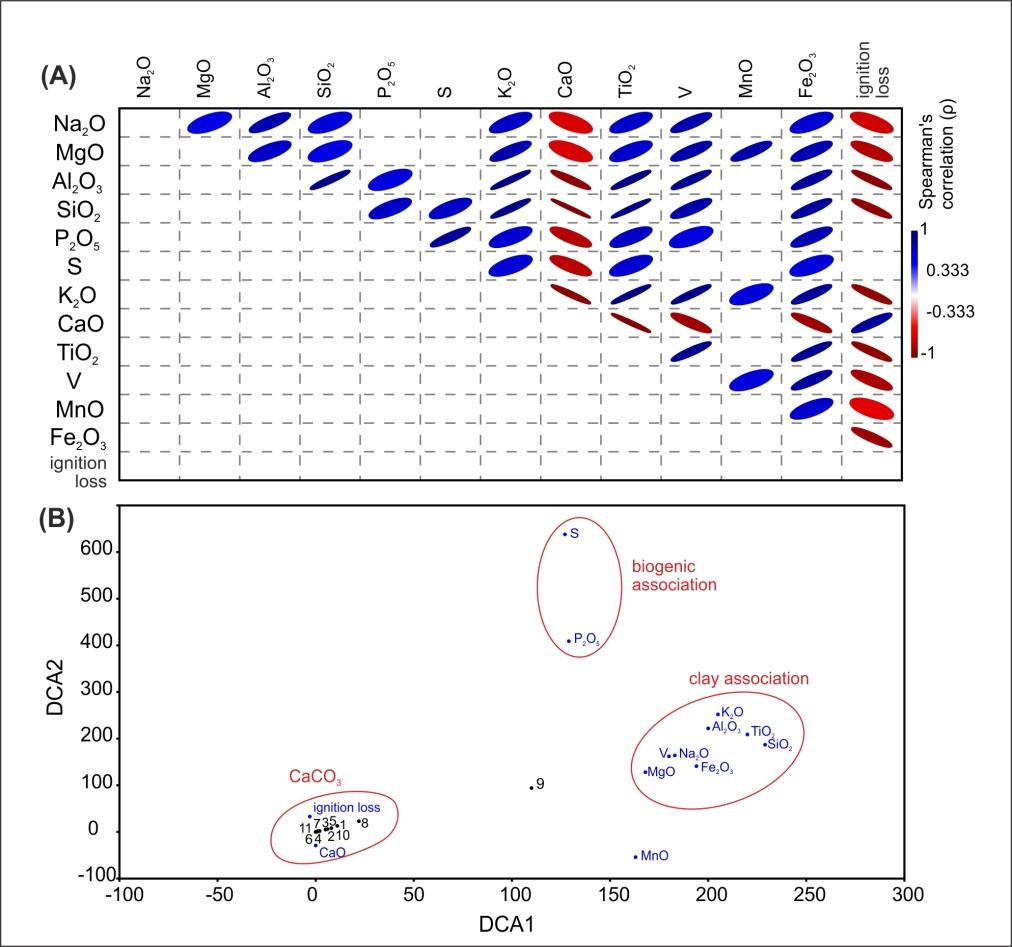


**Fig. S6**. Assessment of the relationship of chemical elements in substrates: a) correlation matrix (Spearman correlation, results shown significant at p < 0.05), b) DCA – density.

**Table S2**. NO_3_ and NO_2_ in drip water, descriptive statistics

| Statistic | NO_3_ | NO_2_ | NO_3_ | NO_2_ |
| --- | --- | --- | --- | --- |
|  | drips under biofilms | | drips in halls without biofilms | |
| Number. of observations | 201 | 196 | 36 | 32 |
| Minimum, mg/l | 0,0 | 0,00 | 0,0 | 0,00 |
| Maximum, mg/l | 22,2 | 0,43 | 6,4 | 0,18 |
| Median, mg/l | 3,7 | 0,00 | 0,2 | 0,00 |
| Mean, mg/l | 4,1 | 0,05 | 0,7 | 0,01 |
| Standard deviation (n), mg/l | 3,4 | 0,09 | 1,3 | 0,04 |
| Variation coefficient (n) | 0,8 | 1,76 | 1,8 | 2,71 |
| Standard error of the mean, mg/l | 0,2 | 0,01 | 0,2 | 0,01 |

**Table S3.** NO_3_ and NO_2_ in drip water, Kruskal-Wallis test / Two-tailed test (drips under biofilms – drips in halls without biofilms)

|  | NO_3_ | NO_2_ |
| --- | --- | --- |
| K (Observed value) | 46,61 | 8,44 |
| K (Critical value) | 3,84 | 3,84 |
| DF | 1 | 1 |
| p-value (one-tailed) | < 0,0001 | 0,004 |
| alpha | 0,05 | 0,05 |
|  |  |  |


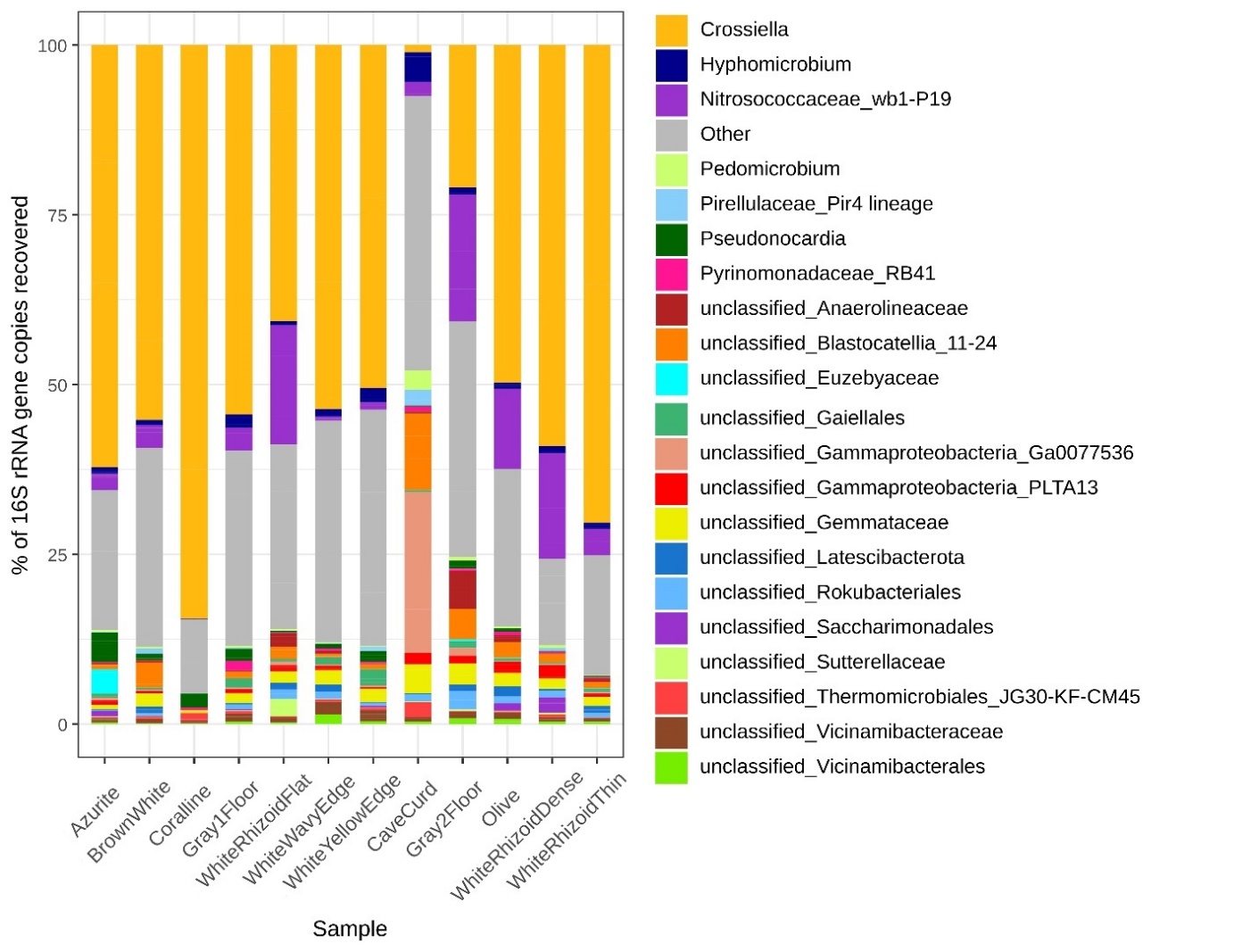


**Figure S7**. Relative abundances of the bacterial genus in the communities of the cave biofilms. Only the genus with the abundance of ≥ 3% in the analyzed samples are present; the other genera are merged into the “Other” category. Classification of the ASVs was conducted against the Silva prokaryotic SSU database (138.1 release) with 50% threshold.


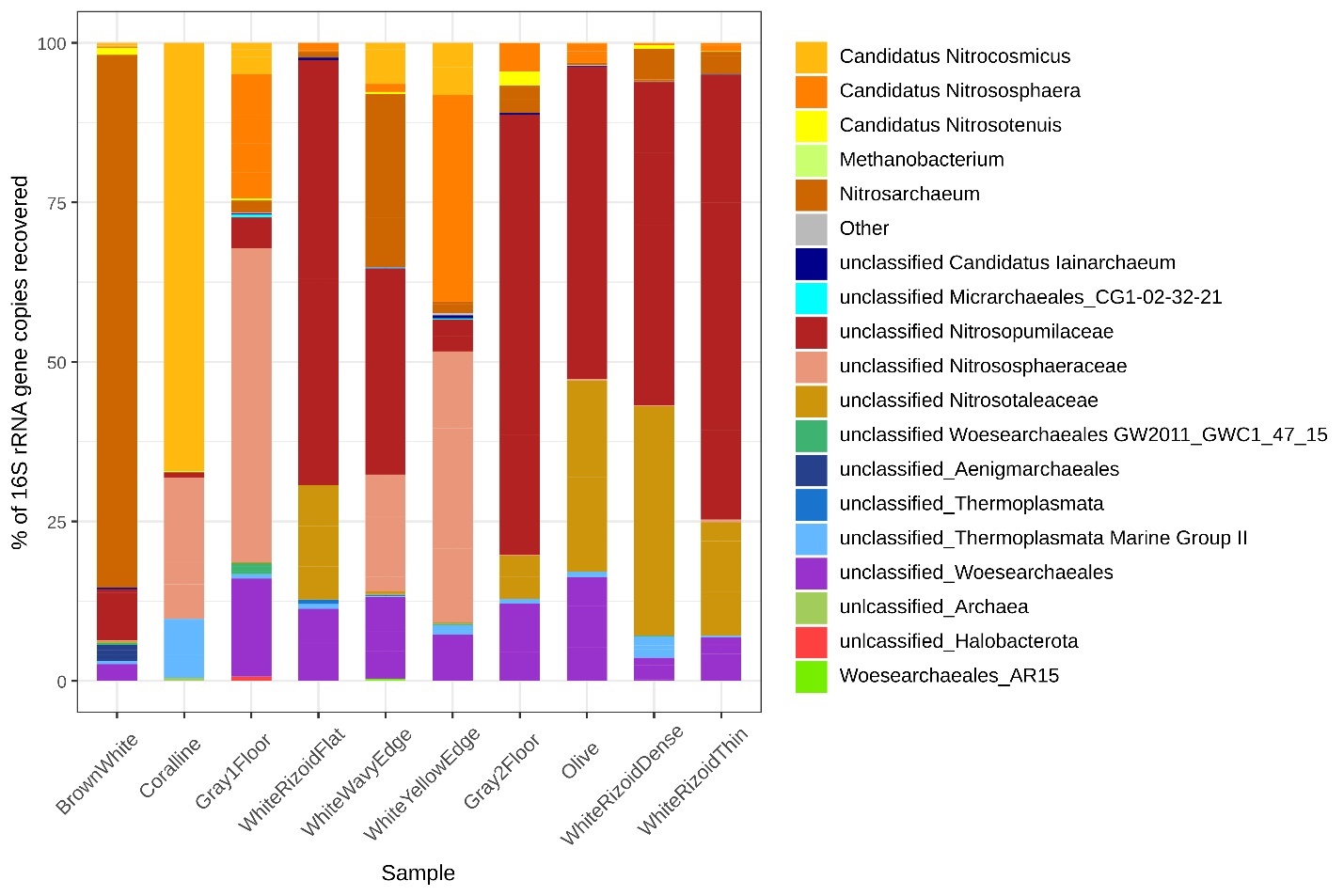


**Figure S8**. The taxonomic composition of the archaea in the cave biofilms at the genus level. Classification of the ASVs was conducted against the Silva prokaryotic SSU database (138.1 release) with 50% threshold.


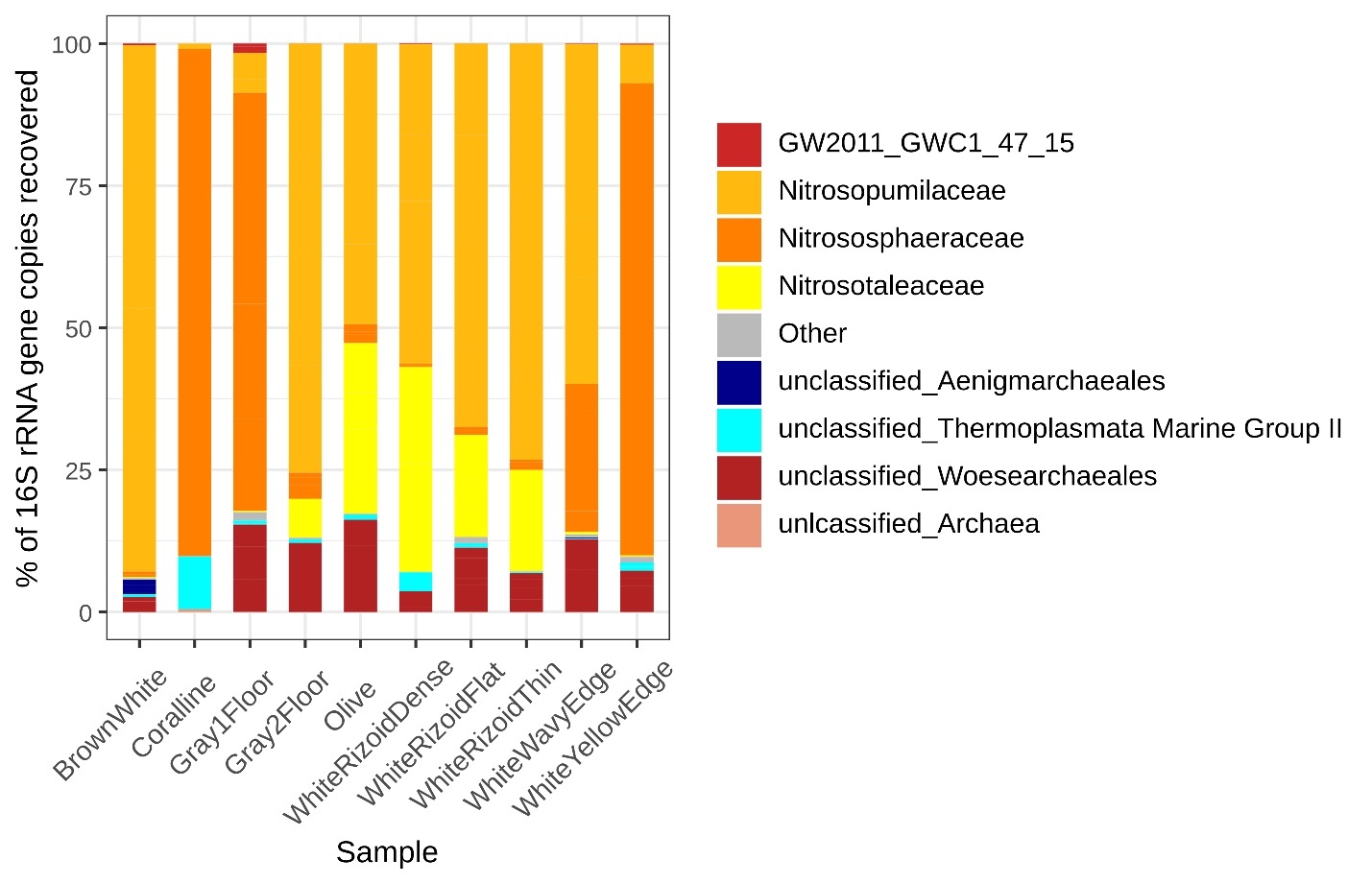


**Figure S9.** The taxonomic composition of the archaea in the cave biofilms at the family level. Only the families with the abundance of ≥ 1% in the analyzed samples are present; the other families are merged into the category “Other”. Classification of the ASVs was conducted against the Silva prokaryotic SSU database (138.1 release) with 50% threshold.


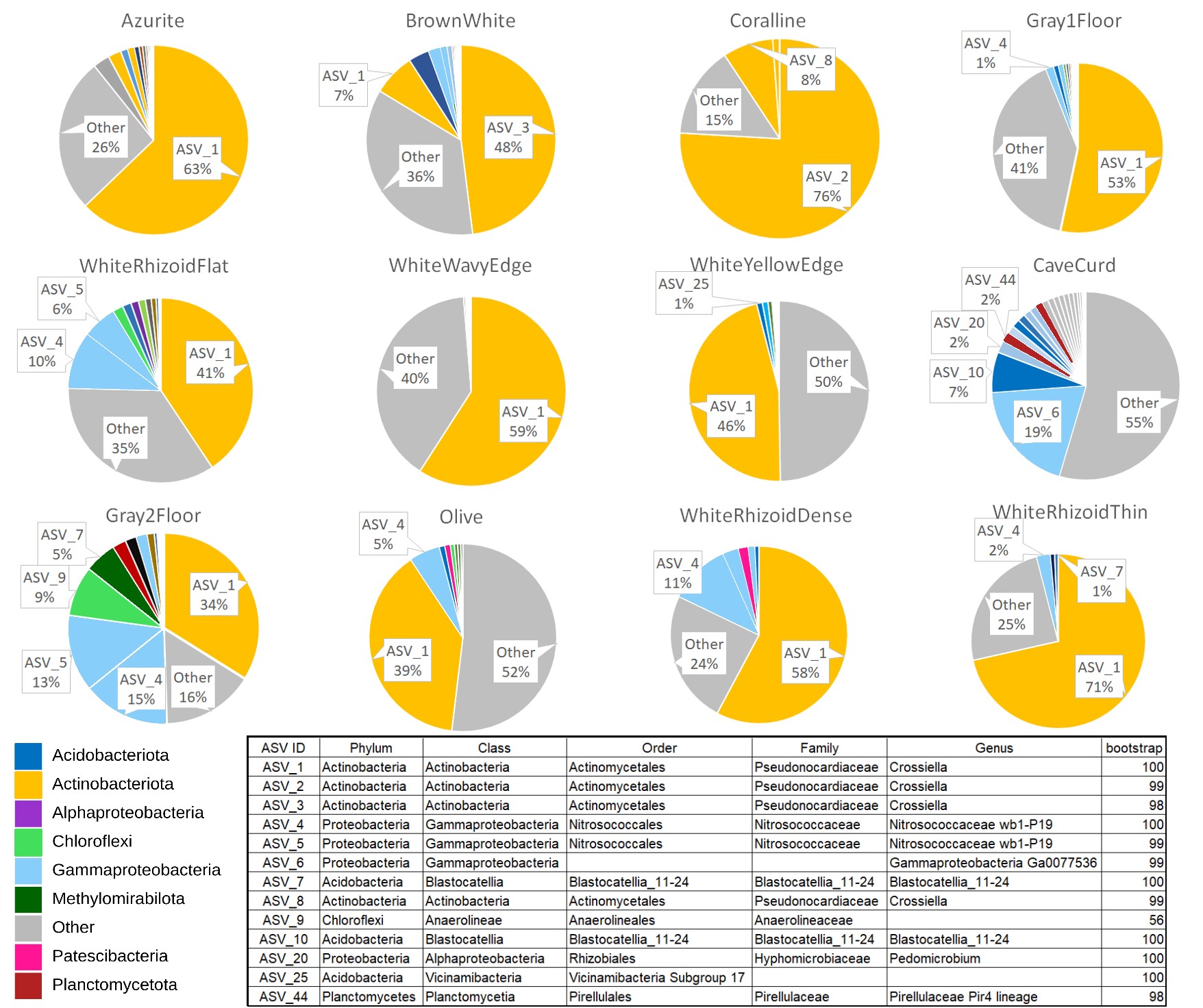


**Figure S10.** Representation of dominant bacterial taxa in the biofilms of the Shulgan-Tash cave. The pie charts show the ratio of taxa in the microbial community of the biofilms. The shares of the ASVs represented by more than 0.5% are highlighted in colors. The table provides a taxonomic annotation of the most represented ASVs, indicating the level of taxon identification.


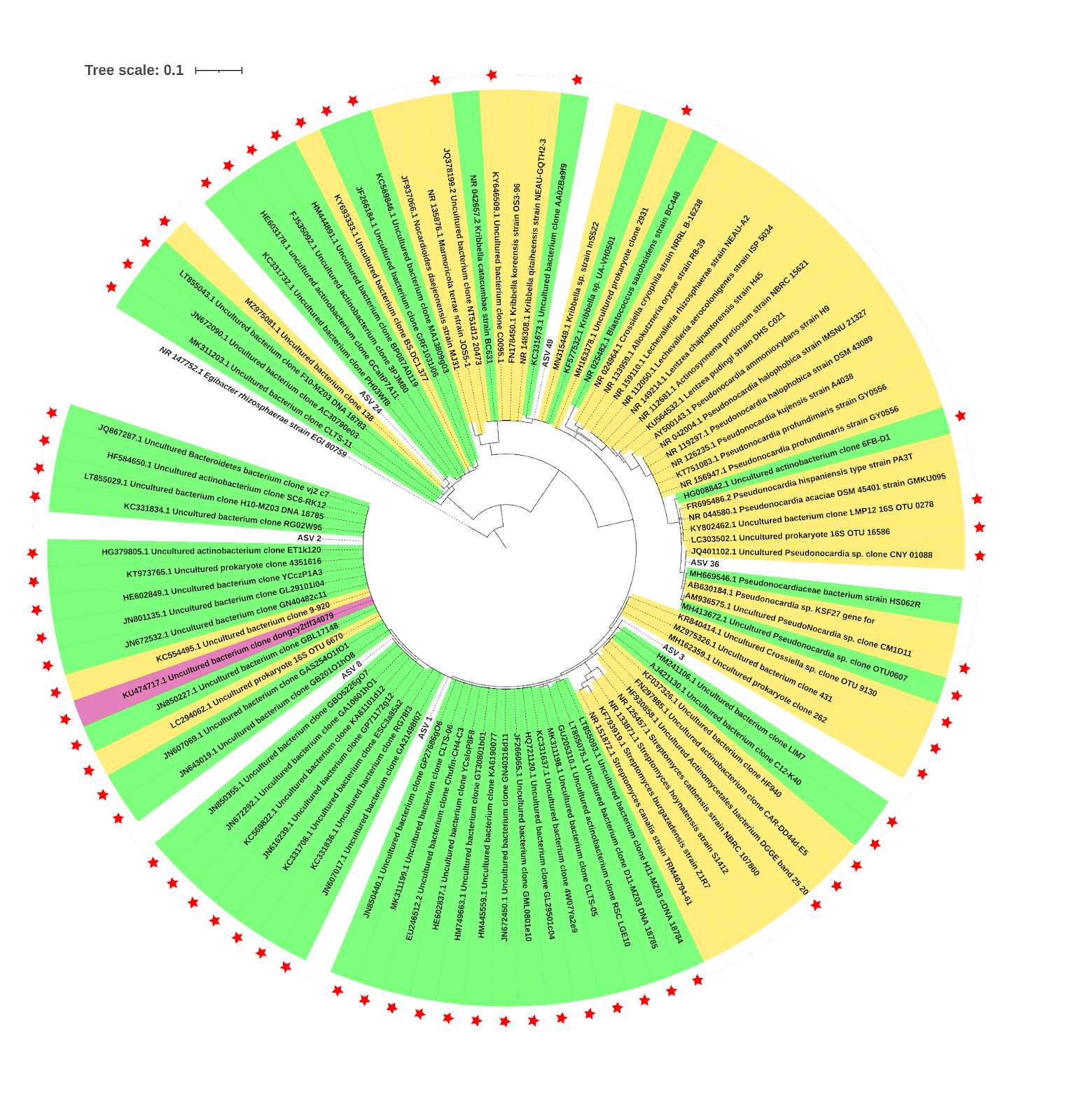


**Figure S11**. Rooted maximum likelihood RaxML phylogenetic tree based on V4 region of 16S rRNA genes showing the relationships of *Actinobacteria* members across different sample locations. ASVs detected in Shulgan-Tash cave and outgroup sequences were not highlighted with color. Sequences isolated from caves, soils and hot springs highlighted with green, yellow and purple respectively. Sequences of uncultivated bacteria were marked with asterisks.


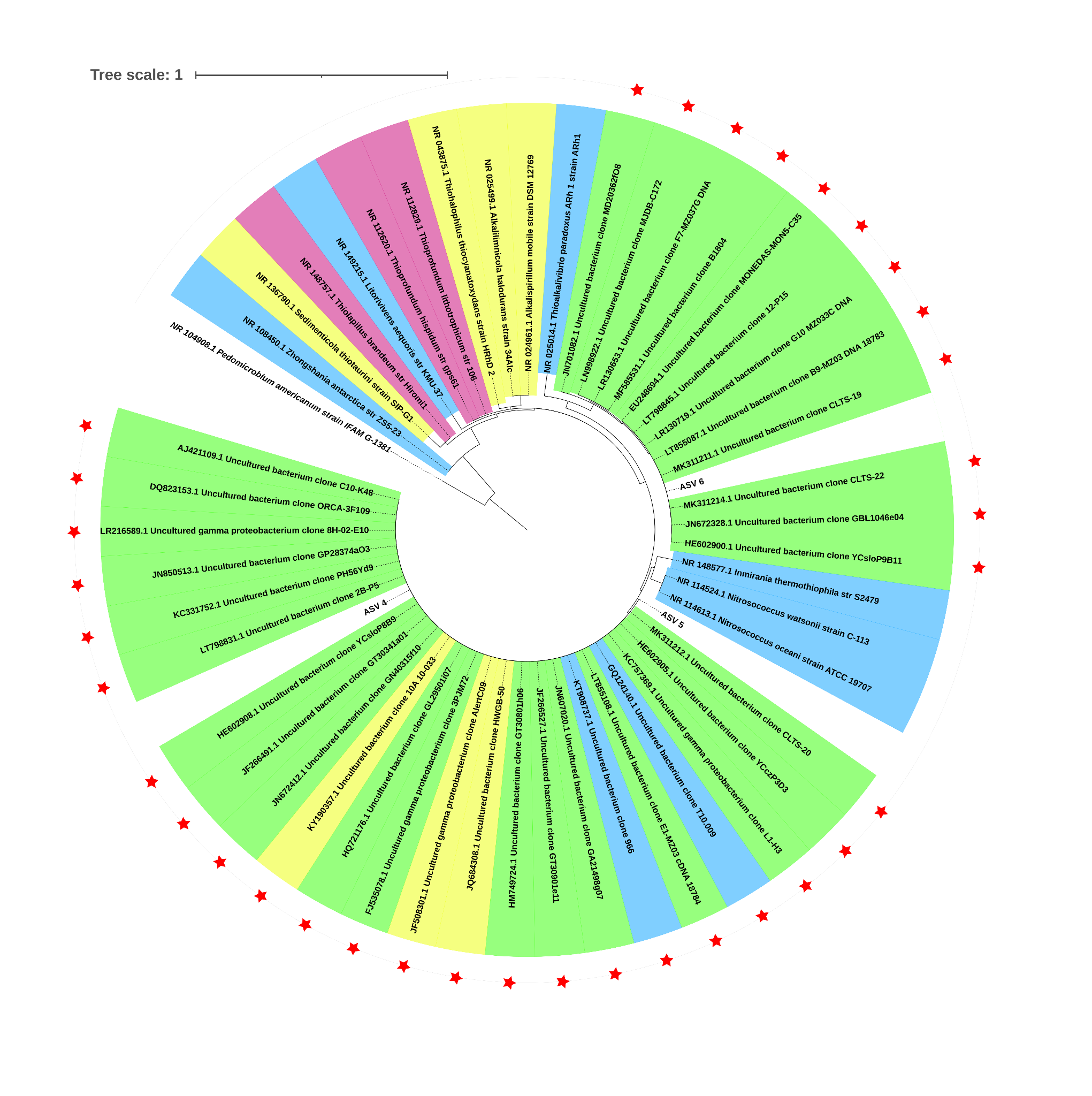


**Figure S12.** Rooted maximum likelihood RaxML phylogenetic tree based on V4 region of 16S rRNA genes showing the relationships of the order Ga0077536 members across different sample locations. ASVs detected in Shulgan-Tash cave and outgroup sequences were not highlighted with color. Sequences isolated from caves, soils, water sources, and hot springs highlighted with green, yellow, blue, and purple respectively. Sequences of uncultivated bacteria were marked with an asterisk.


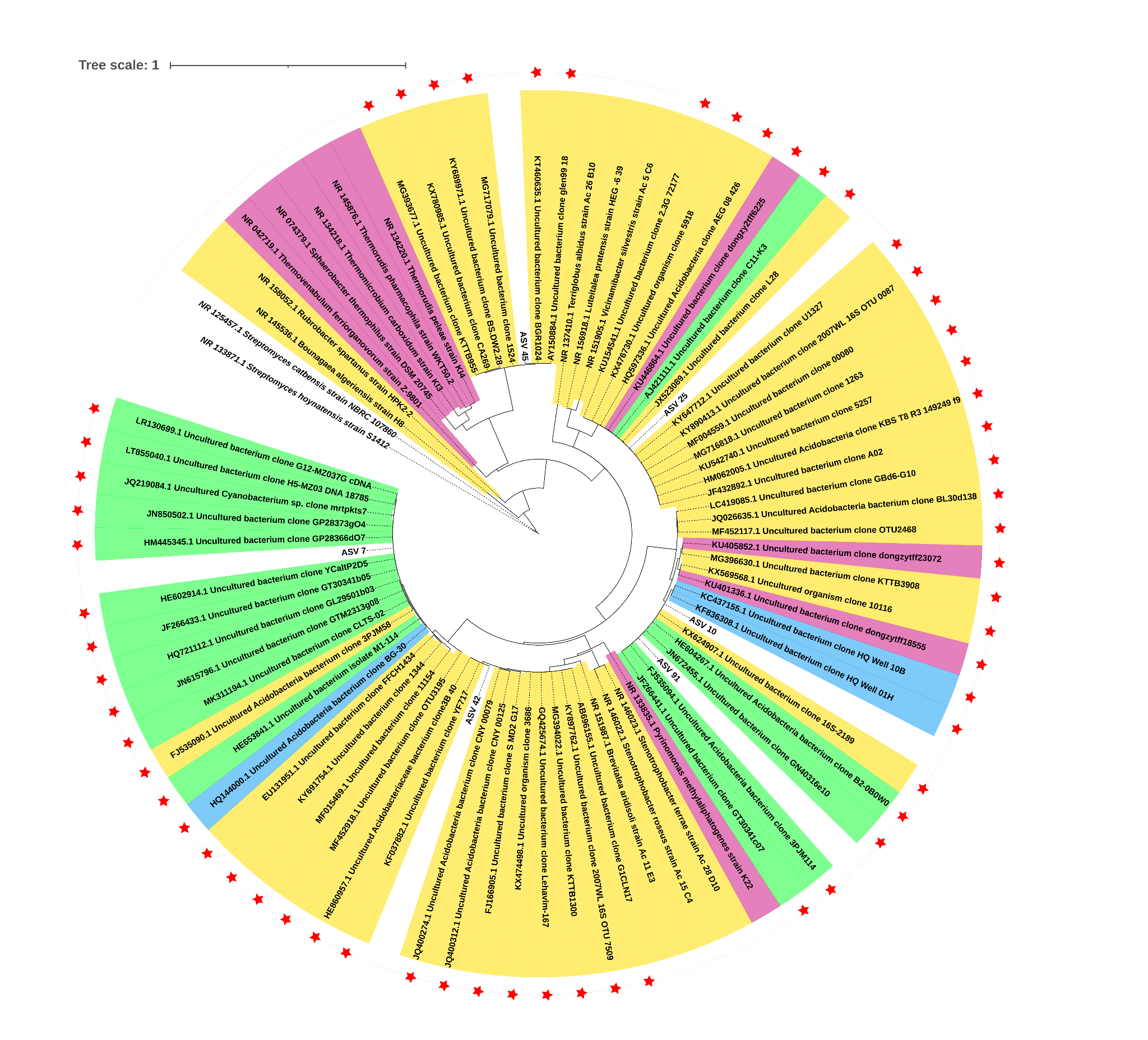


**Figure S13**. Rooted maximum likelihood RaxML phylogenetic tree based on V4 region of 16S rRNA genes showing the relationships of *Acidobacteria* members across different sample locations. ASVs detected in Shulgan-Tash cave and outgroup sequences were not highlighted with color. Sequences isolated from caves, soils, water sources, and hot springs highlighted with green, yellow, blue, and purple respectively. Sequences of uncultivated bacteria were marked with an asterisk.


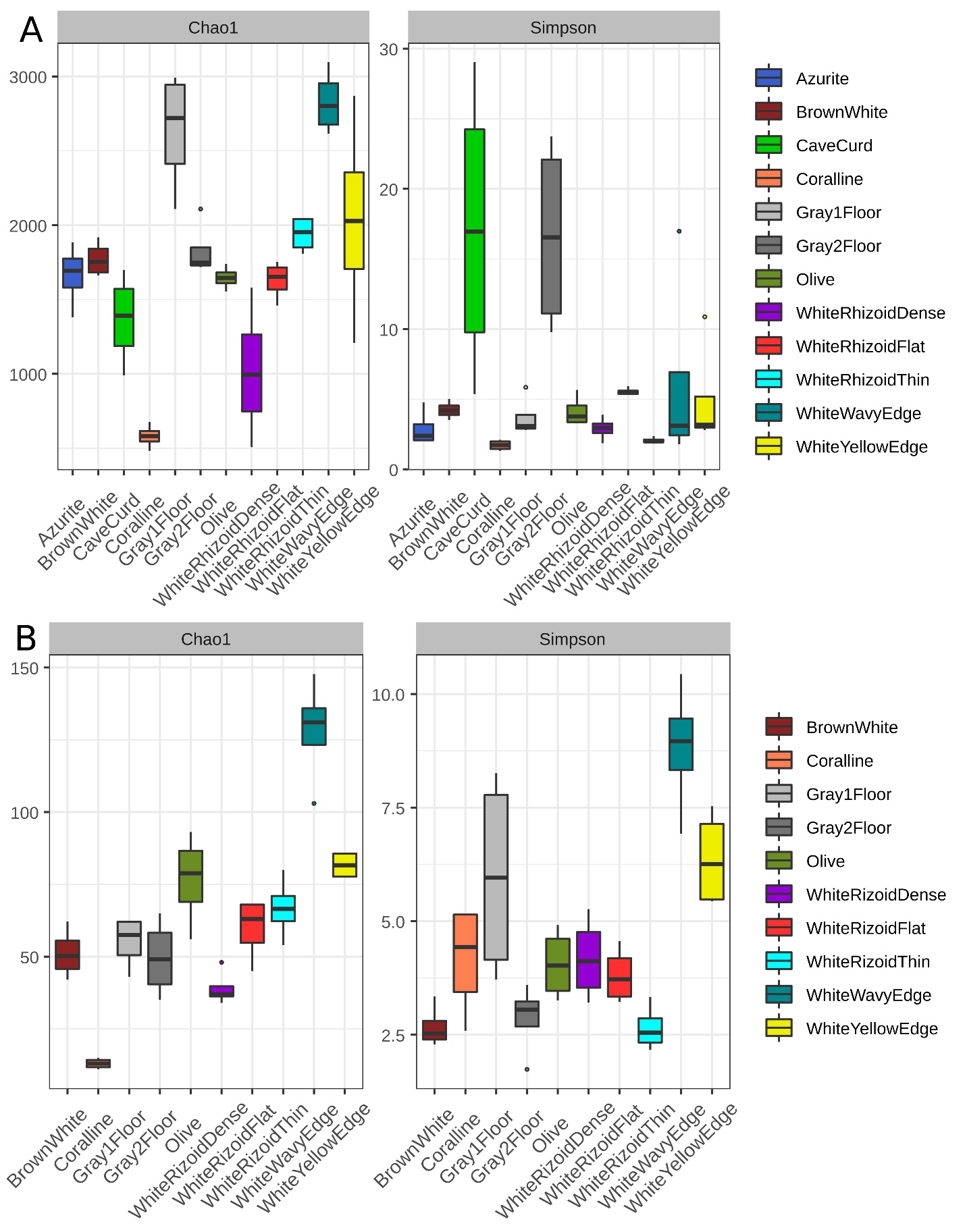


**Figure S14**. Alpha diversity indices (Chao1 and Inverted Simpson) of bacterial (A) and archaeal (B) communities of the Shulgan-Tash cave.


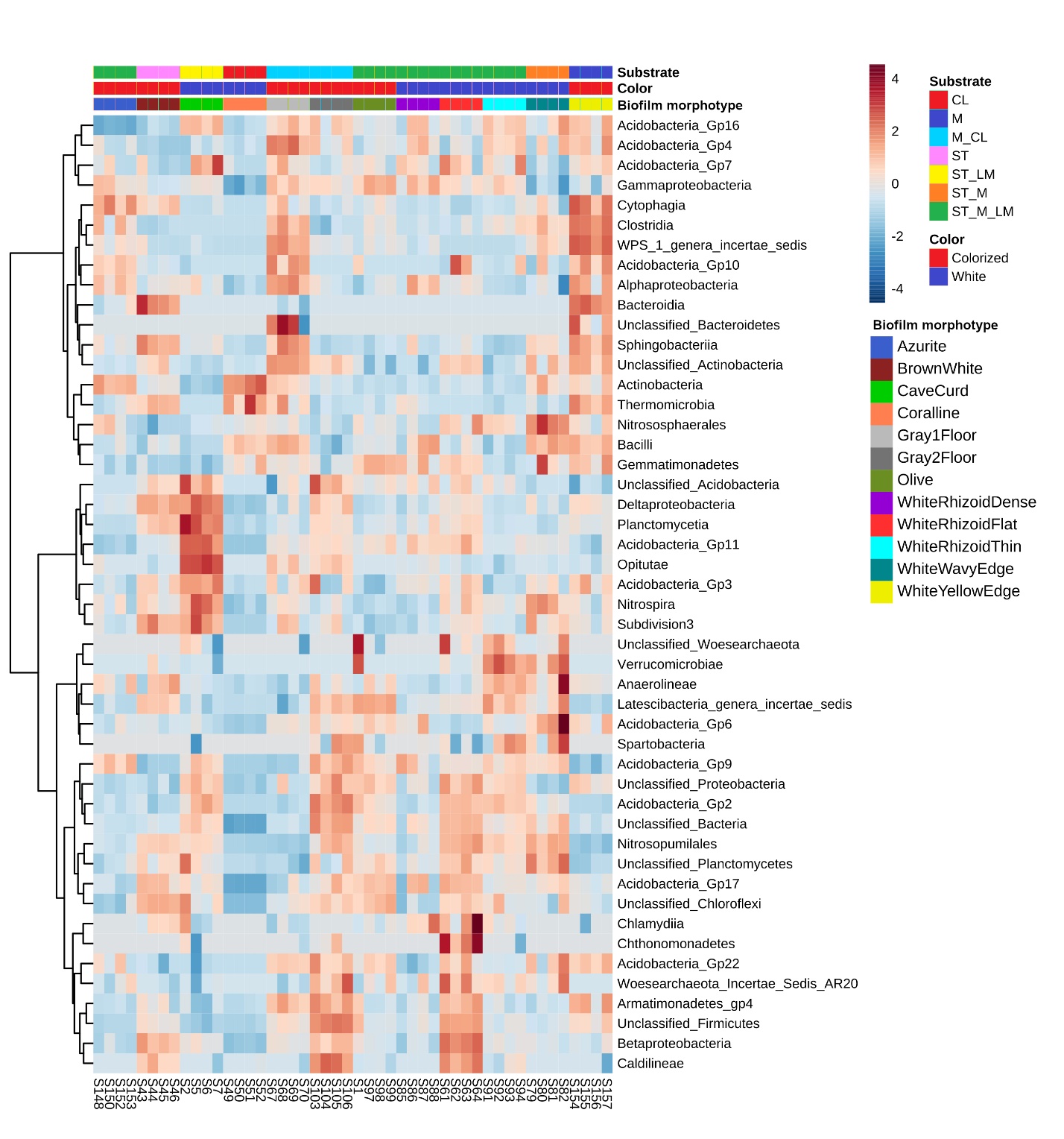


**Figure S15**. Heat map, showing taxonomy composition of the bacterial communities of cave biofilms at the class level. Centered log ratio (CLR) transformation was applied for data normalization.
